# Predicting Immunotherapy Outcomes in NSCLC Using RNA and Pathology from Multicenter Clinical Trials

**DOI:** 10.1002/advs.202502037

**Published:** 2025-10-29

**Authors:** Zhaojun Wang, Yiran Fang, Xiatong Huang, Guichuang Ma, Qianqian Mao, Xiansheng Lu, Guangda Rong, Yunfang Yu, Yuanyuan Wang, Zhenhua Huang, Huiying Sun, Jiani Wu, Wenchao Gu, Na Huang, Jianhua Wu, Rui Zhou, Xiaoxiang Rong, Siting Zheng, Shaowei Li, Gaofeng Wang, Ling Wang, Wenjun Qiu, Luyang Jiang, Peng Luo, Yonggang Liu, Jianping Bin, Yulin Liao, Min Shi, Zuqiang Wu, Jiguang Wang, Wangjun Liao, Gang Chen, Dongqiang Zeng

**Affiliations:** ^1^ Department of Oncology Nanfang Hospital Southern Medical University Guangzhou Guangdong 510515 P. R. China; ^2^ Cancer Center the Sixth Affiliated Hospital School of Medicine South China University of Technology Foshan 528000 China; ^3^ Foshan Key Laboratory of Translational Medicine in Oncology the Sixth Affiliated Hospital School of Medicine South China University of Technology Foshan 528000 China; ^4^ Department of Pathology Nanfang Hospital Southern Medical University Guangzhou Guangdong 510515 P.R. China; ^5^ Department of Medical Oncology Sun Yat‐sen Memorial Hospital Sun Yat‐sen University Guangzhou 510120 China; ^6^ Faculty of Medicine Macau University of Science and Technology Taipa Macao 999078 China; ^7^ Department of Artificial Intelligence Medicine Graduate School of Medicine Chiba University Chiba 260‐867 Japan; ^8^ Department of Plastic and Aesthetic Surgery Nanfang Hospital of Southern Medical University Guangzhou Guangdong 510515 China; ^9^ Department of Dermatology Johns Hopkins University School of Medicine Baltimore MD 21210 USA; ^10^ Department of Oncology Zhujiang Hospital Southern Medical University Guangzhou Guangdong 510280 China; ^11^ Department of Oncology The Eighth Affiliated Hospital of Southern Medical University (The First People's Hospital of Shunde, Foshan) Foshan Guangdong 528308 China; ^12^ Department of Cardiology Nanfang Hospital Southern Medical University Guangzhou Guangdong 510515 P.R. China; ^13^ Division of Life Science Department of Chemical and Biological Engineering Centre of Systems Biology and Human Health and State Key Laboratory of Molecular Neuroscience The Hong Kong University of Science and Technology Hong Kong 999077 P. R. China; ^14^ Department of Medical Oncology Sun Yat‐sen University Cancer Center State Key Laboratory of Oncology in South China Collaborative Innovation Center for Cancer Medicine 510060 Guangdong Province China

**Keywords:** artificial intelligence (AI), immune checkpoint inhibitors (ICIs), immunotherapy response, machine learning, non‐small cell lung cancer (NSCLC)

## Abstract

Immune checkpoint inhibitors (ICIs) are widely used to treat advanced non‐small cell lung cancer (NSCLC). However, it remains crucial to identify patients who are unlikely to benefit from immunotherapy and to explore potential combination treatment strategies. In this study, 1127 advanced NSCLC patients from multicenter randomized clinical trials (OAK, POPLAR, ORIENT‐11) and an in‐house cohort who received ICIs, ICIs combined with chemotherapy, or chemotherapy alone are analyzed. Using bulk RNA‐seq transcriptomic data, an RNA‐based model, named the Lung Cancer Immunotherapy Response Assessment (LIRA), is developed, utilizing interaction analysis and a random forest algorithm to predict immunotherapy outcomes. LIRA outperforms PD‐L1 expression and tumor mutation burden in predicting responses, particularly in identifying early progression risk during ICI monotherapy (HR: 0.15, 95% CI: 0.11–0.20). Tumor profile analysis reveals that LRP8 and HDAC4 are associated with immunotherapy outcomes. Additionally, scRNA‐seq analysis of NSCLC tumors indicates a higher prevalence of T cells and a reduced proportion of epithelial cells in samples with a high LIRA‐score. The deep learning model pinpointed critical high‐attention regions within whole‐slide images that contributed decisively to the LIRA predictions. In summary, these results demonstrate that LIRA enables independent risk stratification of NSCLC patients and provides insights into potential resistance mechanisms.

## Graphical abstract

LIRA, a machine learning‐based model, is developed using transcriptomic data from 891 NSCLC patients in the OAK and POPLAR cohorts. Its predictive performance is validated in multiple external cohorts. Patients stratified by LIRA‐score exhibit distinct clinical characteristics and tumor microenvironment profiles. Additionally, a deep learning model enables LIRA‐score prediction directly from whole‐slide pathology images.

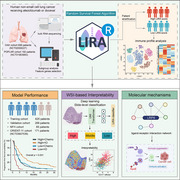

## 1 Introduction

For patients with advanced non‐small cell lung cancer (NSCLC), immune checkpoint inhibitor (ICI) monotherapy or combination chemotherapy with ICIs has become the standard first‐line treatment for patients not harboring driver alterations.^[^ ^1^, ^2^ ^]^ Several phase III trials have reported that patients receiving ICI treatment exhibited significantly prolonged median overall survival (OS) compared to those receiving chemotherapy.^[^ ^3^, ^4^ ^]^ However, we still lack reliable biomarkers to predict the survival benefit of ICIs.^[^ ^5^ ^]^

The most widely used biomarker for ICI therapy is programmed death ligand‐1 (PD‐L1) expression on tumor cells.^[^ ^6^, ^7^ ^]^ Previous studies have demonstrated a positive correlation between PD‐L1 expression and the prognosis of patients receiving immunotherapy.^[^ ^8^, ^9^ ^]^ Another well‐established biomarker for predicting tumor response to ICI treatment is tumor mutation burden (TMB), and high levels have been reported as a favorable prognostic biomarker.^[^ ^10^, ^11^ ^]^ Additionally, tumor‐infiltrating lymphocytes (TILs) or T cell‐inflamed gene expression profiles (GEP)^[^ ^12^ ^]^ have been reported as predictors of the immunotherapy response in clinical trials. However, the predictive power of these biomarkers is limited by the complexity and heterogeneity of the tumor microenvironment (TME), making it difficult to accurately identify patients who will benefit from immunotherapy.^[^ ^13^, ^14^, ^15^, ^16^, ^17^ ^]^ Consequently, there is an urgent need to explore alternative biomarkers capable of accurately predicting the response to immunotherapy in patients.

Artificial intelligence (AI) has shown significant promise for assisting in advanced NSCLC. It has been broadly applied in diagnosis, prognosis, and adverse effect prediction.^[^ ^18^ ^]^ AI‐based tools, capable of analyzing complex biological patterns of tumors, offer a significant advantage over traditional methods by overcoming the constraints of manual scoring and human bias. As a subset of AI, machine learning is defined as a method of analyzing extensive sample data and parsing that data into predictive models.^[^ ^19^ ^]^ Previous studies have confirmed the efficacy of radiomics‐based AI models in predicting responses to immunotherapy in patients with advanced NSCLC.^[^ ^20^, ^21^ ^]^ However, there is still a notable absence of AI models based on transcriptome data that can accurately predict the response to ICIs.^[^ ^22^ ^]^ Consequently, this study aimed to create a machine learning model to accurately predict a patient's probability of immunotherapy response by comprehensively selecting feature genes associated with immunotherapy efficacy based on a large cohort with ICI therapy.

To achieve precise prediction of survival benefits to ICI treatment, we developed a machine learning‐based assessment model called the Lung cancer Immunotherapy Response Assessment (LIRA), employing a random survival forest method based on the transcriptomes of 891 patients with advanced NSCLC receiving atezolizumab monotherapy or docetaxel from POPLAR^[^ ^23^ ^]^ (phase II) and OAK^[^ ^24^ ^]^ (phase III) trials. LIRA computes a LIRA‐score from bulk RNA‐seq data, serving as a biomarker for immunotherapy benefit. The predictive value of LIRA was investigated in the POPLAR, OAK, ORIENT‐11^[^ ^25^ ^]^ and in‐house Nanfang Hospital (NFH) cohorts, all treated with ICIs (PD‐1 or PD‐L1 blockade), ICI combination therapy, or chemotherapy alone. To delineate the immune profiles of tumors with distinct LIRA‐score, we analyzed single‐cell RNA sequencing (scRNA‐seq) data comprising 41799 cells from an independent NSCLC cohort. We also used an attention‐based deep learning model to identify regions on whole slide images (WSIs) that contribute to the prediction of LIRA‐score.

## 2 Results

### 2.1 LIRA Predicts Survival Outcomes of PD‐L1 Blockade Monotherapy in Second‐Line or Third‐Line Treatment

**Table** **1** and Tables S1–S3 (Supporting Information) summarize the demographic variables and clinical characteristics of the training cohort, internal validation cohort, and external validation cohorts (in‐house NFH cohort and ORIENT‐11 cohort).^[^ ^23^, ^24^, ^25^ ^]^ The general clinical information, including gender, histopathologic subtype, and driver mutation subtype, was well matched between the immunotherapy and chemotherapy groups within cohorts.

**Table 1:** Baseline characteristics of the patients according to the therapy in four NSCLC cohorts.

	POPLAR cohort		OAK cohort		ORIENT‐11 cohort		NFH cohort
	IO (*n* = 95)	Chemo (*n* = 97)	IO (*n* = 344)	Chemo (*n* = 355)	Combo (*n* = 113)	Chemo (*n* = 58)	Combo (*n* = 65)
**LIRA‐group,** *n* **[%]**							
High	45 (47.4%)	40 (41.2%)	156 (45.3%)	149 (42.0%)	85 (75.2%)	40 (69.0%)	36 (55.4%)
Low	50 (52.6%)	57 (58.8%)	188 (54.7%)	206 (58.0%)	28 (24.8%)	18 (31.0%)	29 (44.6%)
**gender,** *n* **[%]**							
Female	27 (28.4%)	44 (45.4%)	125 (36.3%)	133 (37.5%)	26 (23.0%)	13 (22.4%)	12 (20.0%)
Male	68 (71.6%)	53 (54.6%)	219 (63.7%)	222 (62.5%)	87 (77.0%)	45 (77.6%)	48 (80.0%)
**BOR,** *n* **[%]**							
CR	1 (1.05%)	0 (0.0%)	4 (1.16%)	0 (0.0%)	3 (2.65%)	2 (3.45%)	0 (0.0%)
PR	12 (12.6%)	16 (16.5%)	44 (12.8%)	42 (11.8%)	73 (64.6%)	21 (36.2%)	25 (38.5%)
SD	40 (42.1%)	35 (36.1%)	111 (32.3%)	158 (44.5%)	24 (21.2%)	24 (41.4%)	32 (49.2%)
PD	34 (35.8%)	35 (36.1%)	159 (46.2%)	115 (32.4%)	13 (11.5%)	9 (15.5%)	8 (12.3%)
NE	8 (8.42%)	11 (11.3%)	26 (7.56%)	40 (11.3%)	0 (0.0%)	2 (3.45%)	0 (0.0%)
**HIST,** *n* **[%]**							
Non‐sq	59 (62.1%)	61 (62.9%)	257 (74.7%)	252 (71.0%)	113 (100%)	58 (100%)	45 (69.2%)
Sq	36 (37.9%)	36 (37.1%)	87 (25.3%)	103 (29.0%)	0 (0.0%)	0 (0.0%)	20 (30.8%)
**TPS,** *n* **[%]**							
[50^,^100]	0 (0.0%)	0 (0.0%)	40 (11.6%)	39 (11.0%)	49 (43.4%)	26 (44.8%)	19 (29.2%)
[1,50)	0 (0.0%)	0 (0.0%)	55 (16.0%)	50 (14.1%)	33 (29.2%)	13 (22.4%)	18 (27.7%)
[0,1)	0 (0.0%)	0 (0.0%)	85 (24.7%)	92 (25.9%)	30 (26.5%)	17 (29.3%)	12 (18.5%)
NE	95 (100%)	97 (100%)	164 (47.7%)	174 (49.0%)	1 (0.88%)	2 (3.45%)	16 (24.6%)
**(t)TMB,** *n* **[%]**							
<16	0 (0.0%)	0 (0.0%)	157 (45.6%)	166 (46.8%)	0 (0. 0%)	0 (0.0%)	1 (1.6%)
≥16	0 (0.0%)	0 (0.0%)	51 (14.8%)	51 (14.4%)	0 (0.0%)	0 (0.0%)	32 (49.2%)
NE	95 (100%)	97 (100%)	136 (39.5%)	138 (38.9%)	113 (100%)	113 (100%)	32 (49.2%)

**Abbreviations**: LIRA, Lung cancer Immunotherapy Response Assessment; IO, Immunotherapy; Chemo, Chemotherapy; Combo, combination treatment; HIST, histology; Non‐sq, non‐squamous; Sq, squamous; TPS, PD‐L1 tumor proportion score; (t)TMB, (tissue) tumor mutation burden; MT, mutation; WT, wide type; BOR, best of response; CR, complete response; PR, partial response; SD, stable disease; PD, progressive disease.

**Figure 1:**
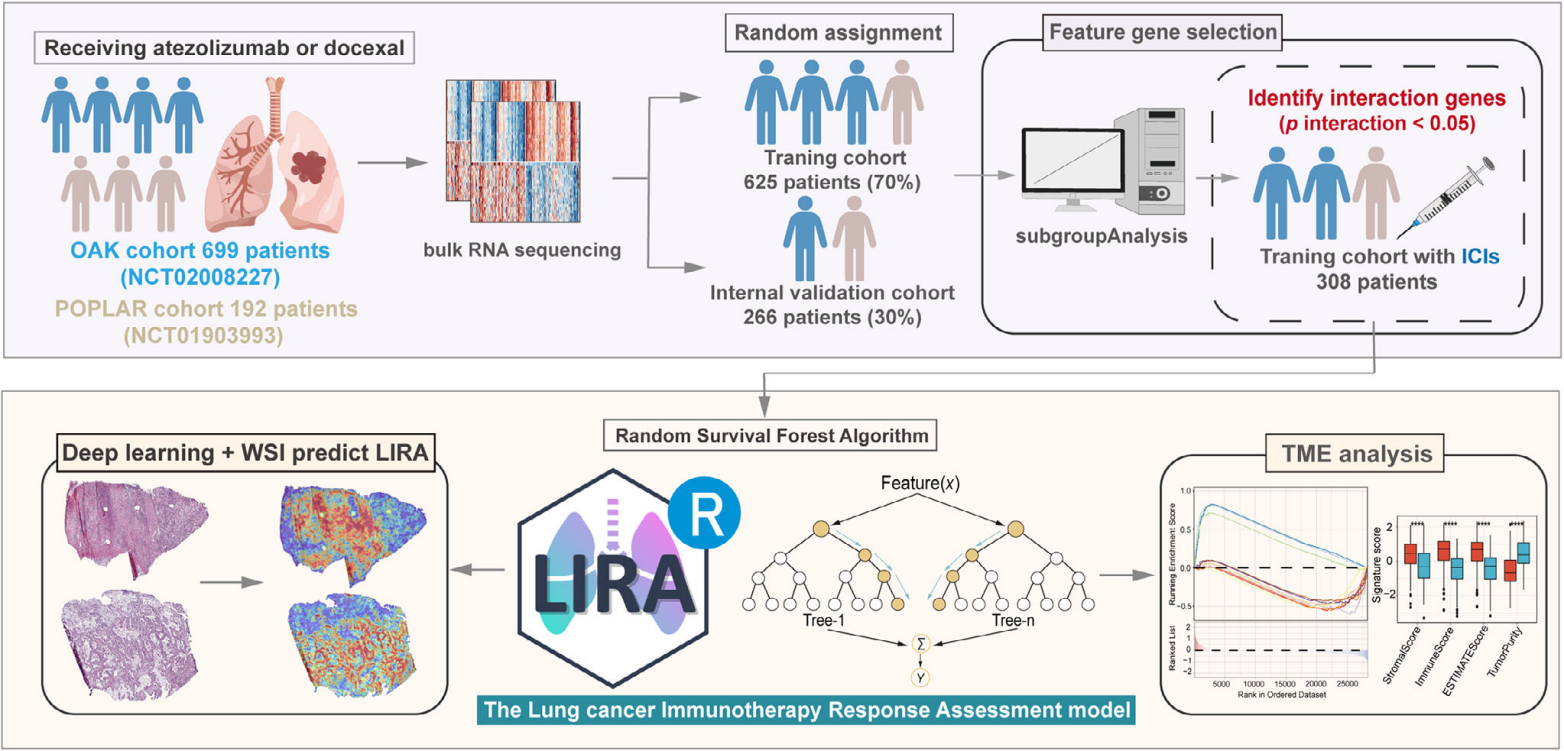
Overview of LIRA development and downstream analysis. Graphical scheme describing the workflow of LIRA development for good response prediction in patients with advanced NSCLC. LIRA, the Lung cancer Immunotherapy Response Assessment; ICIs, immune checkpoint inhibitors; OS, overall survival; PFS, progression‐free survival; TME, tumor microenvironment; IO, immunotherapy; Chemo, chemotherapy.

Based on the transcriptomic data of OAK and POPLAR cohorts,^[^ ^26^, ^27^ ^]^ we developed a predictive model termed LIRA using a random survival forest algorithm to estimate survival outcomes in patients with advanced NSCLC receiving ICI therapy (**Figure** **1**). Gene selection for model construction followed three criteria: 1) genes with *p* < 0.01 in univariate Cox proportional hazards regression analysis, 2) genes showing significant interaction with treatment regimens (docetaxel versus atezolizumab), determined using the “subgroupAnalysis” function of the Publish R package (*p* for interaction < 0.05); 3) the top 50 genes ranked by variable importance (VIMP) in the random survival forest model. The variable importance and hazard ratios (HRs) of these genes for progression‐free survival (PFS) are presented in Figure S1 (Supporting Information). By inputting bulk RNA‐seq data of individual patients, LIRA calculates a corresponding LIRA‐score, which serves as a predictive biomarker for predicting outcome of immunotherapy. The mean LIRA‐score within each cohort was used as a threshold to define patient subgroups. Patients above this threshold (high LIRA‐score group) were predicted to be at low risk, suggesting improved survival benefits (good responders) with ICIs treatment. Conversely, patients below this threshold (low LIRA‐score group) were classified as high‐risk patients who might not achieve the expected survival benefits (poor responders). The training and internal validation cohorts were then divided into low and high LIRA‐score groups for further analysis.

**Figure 2:**
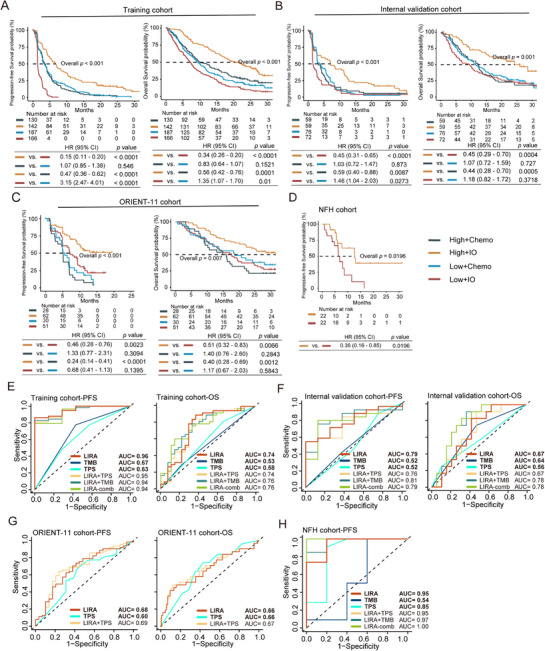
Survival analysis and ROC curves of LIRA in the training and validation cohorts. A–D) Kaplan‐Meier curves comparing PFS and OS among low and high LIRA‐score groups in the training cohort (A), internal validation cohort (B), ORIENT‐11 cohort (C), and NFH cohort (D). E–G) ROC curves and AUC of LIRA (red), tTMB/TMB (dark blue), TPS (cyan), LIRA combined with TPS (light orange), LIRA combined with tTMB/TMB (dark green), and LIRA combined with TPS and tTMB/TMB (leaf green) models for predicting PFS benefit in training cohort (E), internal validation cohort (F), ORIENT‐11 cohort (G), and H) NFH cohort treated with ICIs treatment. HR, hazard ratio; CI, confidence interval; ROC, receiver operating characteristic; AUC, area under the curve; TPS, PD‐L1 tumor proportion score; (t) TMB, (tissue) tumor mutation burden.

The training and internal validation cohorts were derived from the randomized clinical trials POPLAR and OAK, which evaluated the efficacy of atezolizumab versus docetaxel in NSCLC patients who had progressed following platinum‐based chemotherapy.^[^ ^27^ ^]^ We first assessed the predictive performance of LIRA in the training cohort (**Figure** **2**A). Among patients receiving atezolizumab monotherapy, those in the high LIRA‐score group had significantly longer PFS (HR: 0.15, 95% CI: 0.11 – 0.20, *p* < 0.0001) and OS (HR: 0.34, 95% CI: 0.26 – 0.44, *p* < 0.0001), compared to those in the low LIRA‐score group. We then compared survival outcomes between treatment arms within the same LIRA‐score group. In the high LIRA‐score group, patients treated with atezolizumab had significantly better PFS (HR: 0.47, 95% CI: 0.36 – 0.62, *p* < 0.0001) and OS (HR: 0.56, 95% CI: 0.42 – 0.76, *p* < 0.0001) than those treated with docetaxel. In contrast, within the low LIRA‐score group, patients treated with docetaxel had better PFS (HR: 3.15, 95% CI: 2.47 – 4.01, *p* < 0.0001) and OS (HR: 1.35, 95% CI: 1.07 – 1.70, *p* = 0.01) than those treated with atezolizumab (Figure 2A). For both OS and PFS, the area under the curve (AUC) of LIRA exceeded 0.75 at all four time points (6, 12, 18, and 24 months) (Figure S2A, Supporting Information).

We subsequently proceeded to validate the predictive performance of LIRA within an internal validation cohort (*n* = 266; Figure 2B). We first compared survival outcomes between LIRA‐score groups under the same treatment regimen. Within the atezolizumab arm, patients in the high LIRA‐score group showed significantly longer PFS (HR: 0.45, 95% CI: 0.31 – 0.65, *p* < 0.0001) and OS (HR: 0.45, 95% CI: 0.29 – 0.70, *p* = 4×10^−4^) compared to those in the low LIRA‐score group. In contrast, within the docetaxel arm, patients with low LIRA‐score exhibited better PFS than those with high LIRA‐score (HR: 1.46, 95% CI: 1.04 – 2.03, *p* = 0.0273). We then compared outcomes between treatment regimens within the same LIRA‐score group. In the high LIRA‐score group, patients receiving atezolizumab achieved longer PFS (HR: 0.59, 95% CI: 0.40 – 0.88, *p* = 0.0087) and OS (HR: 0.44, 95% CI: 0.28 – 0.70, *p* = 5×10^−4^) than those treated with docetaxel. However, no significant difference in OS was observed in patients with low LIRA‐score (Figure 2B). Time‐dependent receiver operating characteristic (ROC) curves analysis showed the significantly predictive performance of LIRA for the atezolizumab‐treated subgroup was superior to that for OS across multiple time points (Figure S2B, Supporting Information). This indicates that LIRA is more effective at predicting immunotherapy‐associated PFS benefits rather than OS.

In summary, these results suggest that LIRA has a greater capacity to predict PFS benefit from immunotherapy than OS benefit. Patients with high LIRA‐score experience survival benefits from second‐line or later PD‐L1 blockade monotherapy, while patients with low LIRA‐score derive better survival benefits from chemotherapy.

### 2.2 Enhanced Clinical Efficacy of First‐line PD‐1 Blockade Combined with Chemotherapy Correlated with High LIRA‐score

To evaluate the predictive capability of LIRA in patients receiving first‐line PD‐1 inhibitors combined with chemotherapy, we conducted a comprehensive assessment of its performance in the ORIENT‐11 (*n* = 171) and in‐house NFH (*n* = 65) cohorts.^[^ ^25^ ^]^ We first assessed survival outcomes between LIRA‐score subgroups under the same treatment regimen in the ORIENT‐11 cohort (Figure 2C). In the sintilimab plus chemotherapy arm, patients with high LIRA‐score experienced longer PFS (HR: 0.46, 95% CI: 0.28 – 0.76, *p* = 0.0023) and OS (HR: 0.51, 95% CI: 0.32 – 0.83, *p* = 0.0066) compared to those with low LIRA‐score. We then compared survival outcomes across treatment arms within the same LIRA‐score group. In contrast, no statistically significant survival differences were observed between LIRA‐score groups in the chemotherapy arm. Survival outcomes were then compared between treatment regimens within the same LIRA‐score subgroup. Among patients with high LIRA‐score, those receiving sintilimab plus chemotherapy had significantly better PFS (HR: 0.24, 95% CI: 0.14 – 0.41, *p* < 0.0001) and OS (HR: 0.40, 95% CI: 0.28 – 0.69, *p* = 0.0012) compared to those treated with chemotherapy alone. However, in the low LIRA‐score group, no significant differences in PFS or OS were observed between the two treatment strategies.

The predictive performance of LIRA was further validated in the NFH cohort. In the PD‐1 blockade combination therapy arm, patients with high LIRA‐score achieved longer PFS (HR: 0.32, 95% CI: 0.13 – 0.72, *p* = 0.006) compared to low LIRA‐score patients (Figure 2D). Time‐dependent ROC curve analysis confirmed the robust predictive accuracy of LIRA at 6, 12, 18, and 24 months in both the ORIENT‐11 and NFH cohorts (Figure S2C,D, Supporting Information).

To explore LIRA's broader applicability, we next assessed its performance in six non‐ICI NSCLC cohorts (see Methods). LIRA successfully stratified patients in the GSE157001 cohort (postoperative OS) and the TCGA‐LUAD cohort (post‐chemotherapy OS) (Figure S4A–F, Supporting Information), although no statistically significant differences were observed in the remaining four datasets. Additional analyses were performed in other cancer types, including urothelial carcinoma (IMvigor210 cohort) and small cell lung cancer (IMpower133 cohort). In the IMvigor210 cohort, LIRA could predict survival benefit in patients receiving atezolizumab (Figure S4G, Supporting Information). However, no significant survival differences were observed between LIRA‐score groups in the IMpower133 cohort (Figure S4H, Supporting Information).

Taken together, these findings suggest that patients with high LIRA‐score are more likely to benefit from first‐line PD‐1 blockade combination therapy. Conversely, those with low LIRA‐score show limited additional benefit from immunotherapy over chemotherapy, indicating that chemotherapy may be a more cost‐effective option in this subgroup.

### 2.3 Predictive Performance of LIRA is Superior to TPS and TMB

The results above indicate that LIRA effectively identifies advanced NSCLC patients who are likely to benefit from immunotherapy, with superior accuracy in predicting PFS rather than OS. Currently, PD‐L1 expression and TMB remain widely used clinical biomarkers for assessing immunotherapy efficacy in NSCLC. To compare the predictive performance of LIRA with these traditional biomarkers, we performed time‐dependent ROC curve analyses using 12‐month survival as the evaluation time point.

We first assessed the ability of each biomarker to predict PFS benefit in patients receiving ICIs. In the training cohort, LIRA had a higher AUC than PD‐L1 tumor proportion score (TPS; 0.96 versus 0.63, *p* = 0.0015) and tissue TMB (tTMB; 0.96 versus 0.67, *p* = 0.010). Similar results were observed in the internal validation cohort (LIRA versus TPS: 0.78 versus 0.52, *p* = 0.051; LIRA versus tTMB: 0.78 versus 0.52, *p* = 0.0081) for patients treated with atezolizumab (Figure 2E,F; Figure S3A,B, Supporting Information). Similarly, in the ORIENT‐11 cohort, LIRA showed superior predictive value compared to TPS (LIRA versus TPS: 0.68 versus 0.60, *p* = 0.227) in patients receiving PD‐1 blockade plus chemotherapy (Figure 2G; Figure S3D, Supporting Information). Likewise, in the NFH cohort, the predictive performance of LIRA was better than TPS (0.95 versus 0.85, *p* = 0.268) and TMB (0.95 versus 0.54, *p* = 0.072) in the PD‐1 blockade combination therapy arm (Figure 2H; Figure S3C, Supporting Information). We next evaluated the predictive value of these biomarkers for OS. In the training cohort, LIRA performed better than TPS and tTMB (AUC of LIRA = 0.74, AUC of TPS = 0.58, AUC of tTMB = 0.53) (Figure 2E). The internal validation cohort yielded similar results (AUC of LIRA = 0.67, AUC of TPS = 0.56, AUC of tTMB = 0.64) (Figure 2F). However, in the ORIENT‐11 cohort, no significant difference in OS prediction was observed between LIRA and TPS (Figure 2G). Notably, the differences between LIRA and TPS or TMB in the ORIENT‐11 and NFH cohorts did not reach statistical significance, which may be due to the limited number of patients with TPS and TMB data, potentially leading to insufficient statistical power to detect significant differences.

To assess whether combining biomarkers could further enhance predictive accuracy, we constructed Cox proportional hazards models integrating multiple markers. The combination of LIRA and TMB (LIRA–TMB) showed improved performance in the internal validation cohort (AUC for PFS = 0.81; AUC for OS = 0.78) and the NFH cohort (AUC for PFS = 0.97) (Figure 2F,H). The LIRA‐comb (LIRA–TMB–TPS) model achieved the highest PFS AUC (1.00) in the NFH cohort and OS AUC (0.78) in the internal validation cohort. However, the AUC of LIRA‐TPS did not demonstrate a significant improvement compared to LIRA alone in all cohorts. A statistical difference was found between LIRA‐comb model and LIRA (*p* = 0.038). Decision curve analysis (DCA) further supported these findings, indicating that LIRA alone provided a comparable net clinical benefit to the combined models across relevant threshold ranges (Figure S2E–H, Supporting Information).

Overall, LIRA exhibits predictive performance superior to TPS and TMB in patients with ICI treatment. While combining LIRA with other biomarkers may improve the predictive efficacy, no significant differences were observed in statistical comparisons, likely due to the small sample size when multiple markers were evaluated concurrently. Consequently, from an economic standpoint, LIRA alone represents an effective and cost‐efficient tool for predicting immunotherapy outcomes.

### 2.4 LIRA‐Score is Independent of TPS and TMB Levels and Predicts PD‐L1 Blockade Benefit in Patients with Driver Gene Mutations

**Figure 3:**
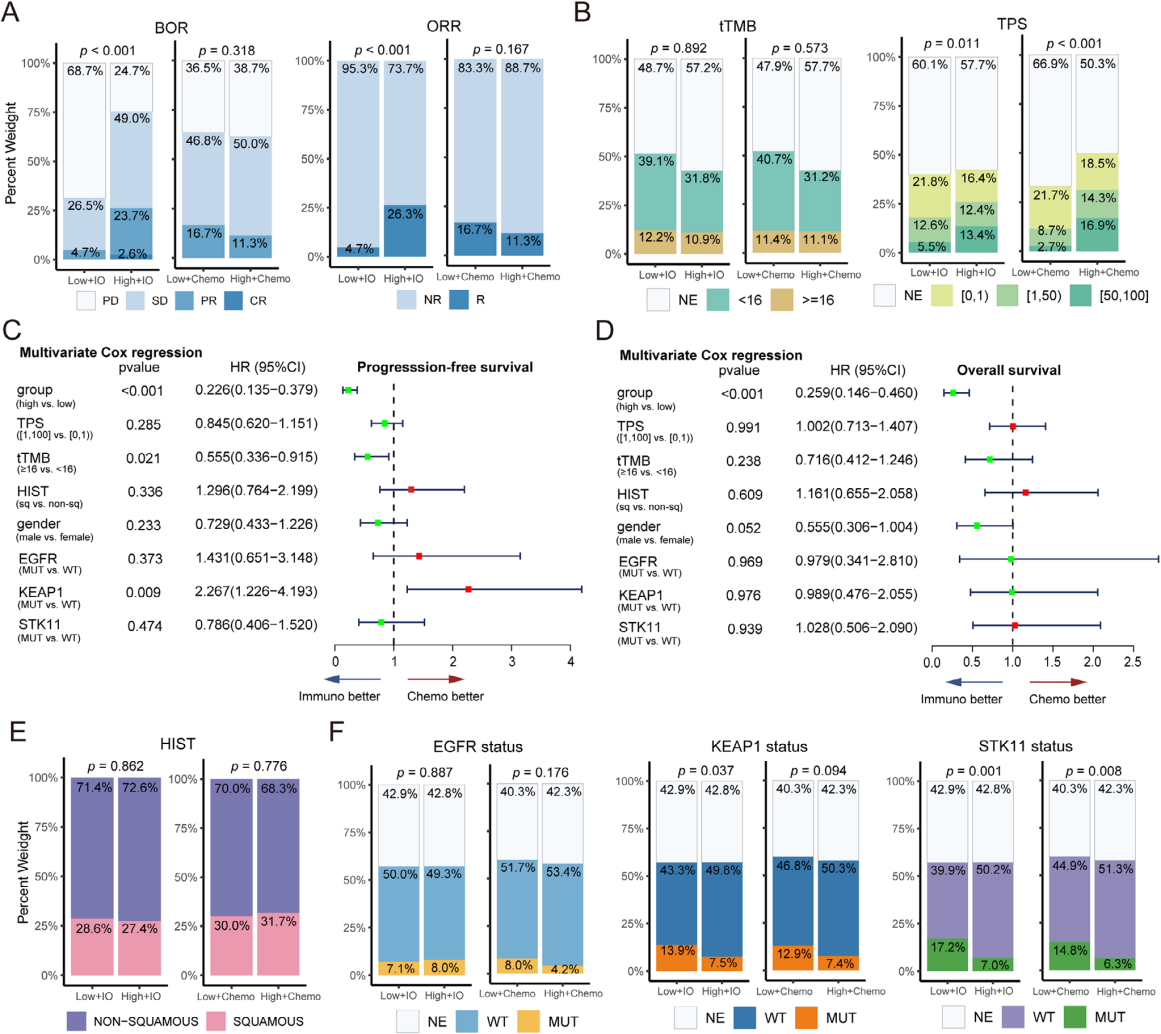
Association of LIRA with clinical features, biomarkers, and mutation status. A,B) Bar plots showing the clinical features and biomarkers, including BOR, ORR, TPS, and tTMB across four groups (*n* = 891). C,D) Forest plots depicting the significance of high LIRA‐score with respect to TPS, tTMB, histology and gender among patients treated with atezolizumab. E,F) Bar plots showing the clinical features and mutation status, including histological subtype and mutation statuses of *STK11*, *KEAP1*, and *EGFR* across four groups (*n* = 891). P values were estimated by means of the Cox model in the multivariate analysis. Hist, histology; BOR, best of response; ORR, objective response rate; CR, complete response; PR, Partial response; SD, Stable disease; PD, Progressive disease; R, response (CR, PR); NR, non‐response (SD, PD); NE, not evaluable; Hist, histology; WT, wild type; MUT, mutation; sq, squamous.

After validating the predictive value of LIRA, we further investigated its relationship with key clinical and molecular characteristics, including *EGFR*, *KEAP1*, *STK11* mutation status, TPS, tTMB, best‐of‐response (BOR), and objective response rate (ORR) across OAK and POPLAR cohorts. Among patients treated with docetaxel, there were no significant differences in BOR or ORR between the low and high LIRA‐score groups (*p* of BOR = 0.318; *p* of ORR = 0.167). In contrast, within the atezolizumab arm, patients with high LIRA‐score achieved better clinical responses (*p* of BOR < 0.001; *p* of ORR < 0.001; **Figure** **3**A), consistent with the results of the survival analysis (Figure 2), further supporting the utility of LIRA‐score as a predictive biomarker for immunotherapy efficacy.

We next explored the association between LIRA‐score and clinical biomarkers, TPS, and TMB. Patients with high LIRA‐score had higher TPS, both in the ICI (*p* = 0.001) and chemotherapy arms (*p* < 0.001) (Figure 3B), suggesting that LIRA‐score may partly reflect PD‐L1 expression level, and that its predictive efficacy could be partially driven by TPS. However, no significant differences in tTMB were detected between LIRA‐score groups (IO: *p* = 0.892; Chemo: *p* = 0.573), indicating that LIRA is likely independent of TMB in predicting treatment response. To evaluate whether LIRA‐score serves as an independent predictive biomarker, multivariate Cox regression analyses were performed. After adjusting for TPS and tTMB, LIRA‐score remained significantly associated with improved survival outcomes (HR for PFS = 0.257, 95% CI: 0.158–0.420, *p* < 0.001; HR for OS = 0.258, 95% CI: 0.147–0.454, *p* < 0.001; Figure 3C,D). Given the clinical relevance of PD‐L1 expression, we further explored the relationship among BOR, LIRA‐score, and TPS (Figure S5A, Supporting Information). Interestingly, some patients with high TPS but low LIRA‐scores did not respond to immunotherapy, while others with low TPS but high LIRA‐score achieved clinical benefit, indicating that LIRA provides prognostic information distinct from TPS and tTMB. The distribution of tissue types was comparable between LIRA‐score groupsc (Figure 3E).

*EGFR*, *KEAP1*, and *STK11* are commonly mutated genes in NSCLC.^[^ ^28^ ^]^ To explore genetic determinants of LIRA‐score, we examined the distribution of *EGFR*, *KEAP1*, and *STK11* mutation statuses between patients with high and low LIRA‐score (Figure 3F). No significant differences were observed in the distribution of *EGFR* mutation status between LIRA‐score groups (IO: *p* = 0.887; Chemo: *p* = 0.176). In contrast, patients in the low LIRA‐score group exhibited a higher proportion of *KEAP1* and *STK11* mutations than those in the high LIRA‐score group (Figure 3F). Notably, we found that *EGFR* mutation was exclusive to *STK11* mutation and *KEAP1* mutation in OAK and POPLAR cohorts (Figure S5B, Supporting Information).

To further assess the impact of these mutations on immunotherapy response, we excluded patients who had not undergone genetic testing and stratified the analyses by mutation status. Among patients with *EGFR* mutations and wild‐type *STK11/KEAP1*, no difference in LIRA‐score distribution was observed (Figure S5C,D, *p* = 0.966, Supporting Information). However, *EGFR*‐mutated patients demonstrated poor immunotherapy response (Figure S5E, Supporting Information), implying that LIRA‐score may predict benefit independently of *EGFR* mutation status. Among patients with *EGFR* wild‐type but mutant *STK11* and/or *KEAP1*, the low LIRA‐score group showed a significantly higher prevalence of these mutations. In particular, 90% of patients with co‐mutations of *KEAP1* and *STK11* fell into the low LIRA‐score group (Figure S5F,G, Supporting Information). Patients harboring co‐mutations of *KEAP1* and *STK11* who received PD‐L1 blockade therapy had poorer survival outcomes than patients with single mutations or wild‐type genes, consistent with findings from previous studies.^[^ ^29^ ^]^ However, this difference did not reach statistical significance (*p* > 0.05; Figure S5H, Supporting Information). To further clarify the predictive value of LIRA across subgroups, we conducted stratified survival analyses based on LIRA‐score and mutation status of *EGFR, STK11* and *KEAP1* in patients treated with atezolizumab. LIRA‐score accurately predicted immunotherapy benefit across all subgroups (Figure S6, Supporting Information). Multivariate Cox analysis further confirmed that LIRA‐score functions as an independent predictive biomarker (Figure 3C,D).

These results indicate that LIRA can identify patients with advanced NSCLC who can benefit from ICIs treatment. Moreover, LIRA can accurately predict the clinical benefit of immunotherapy in patients with different clinical parameters.

### 2.5 Establishing a Reference Database for Single‐Sample Prediction Using LIRA

**Figure 4:**
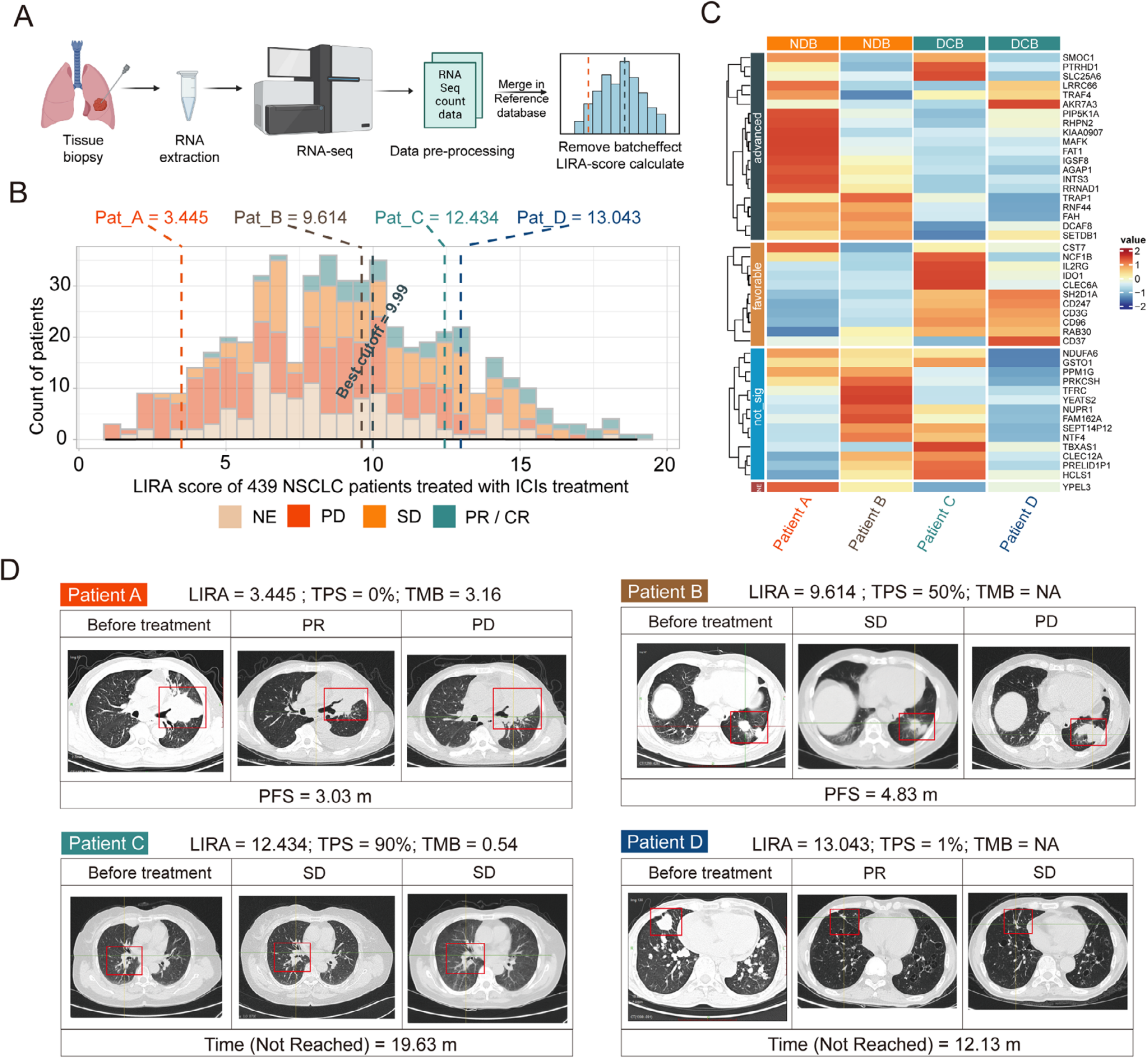
Single‐sample predictive efficacy of LIRA. A) Workflow for calculating single‐sample LIRA‐score using the reference database. B) The histogram shows the distribution of LIRA‐score for four patients (patients A–D). The horizontal axis represents the range of LIRA‐score from 439 advanced NSCLC patients with ICIs treatment in OAK and POPLAR cohorts. C) Heatmap demonstrating the expression of model genes in the four patients. “Advanced” (dark blue) indicates a prognosis associated with poorer outcomes, and “favorable” (brown) suggests a better prognosis. D) CT images showcase tumor development in four patients. The red box marks the location of the tumor. Pat, patient; DCB, durable clinical benefit (DCB; CR/PR or SD that lasted > 6 months); NDB, no durable benefit (PD or SD that lasted ≤ 6 months). Figure (A) was created with BioRender.com and is used with permission.

Upon confirming the predictive ability of LIRA across the cohorts, we next evaluated its potential for individualized prediction by testing its applicability to single samples and small independent cohorts. To ensure comparability between LIRA‐score and the predefined cutoff value, we established a LIRA reference database using transcriptomic data from atezolizumab‐treated patients (*n* = 439) in the OAK and POPLAR cohorts (**Figure** **4**A). Individual bulk RNA‐seq data were integrated into this reference database, followed by batch‐effect removal and LIRA‐score computation to predict the clinical benefit from immunotherapy. The optimal cutoff value for LIRA‐score was determined using the “best_cutoff” function from the IOBR R package^[^ ^30^ ^]^ For single‐sample validation, clinical outcomes were evaluated based on RECIST (v1.1) criteria in combination with durable clinical benefit (DCB) or no durable benefit (NDB) (see Methods).

We selected four patients (A, B, C, and D) from the NFH cohort for case‐based analysis. All four patients had measurable lesions before and after treatment and available TPS data, enabling comparison between multiple biomarkers (Figure 4B). The optimal LIRA‐score cutoff was 9.99. Patients A (TPS = 50%) and B (TPS < 1%) had LIRA‐scores below this threshold (LIRA‐score A = 9.614, LIRA‐score B = 3.445), while patients C (TPS = 90%) and D (TPS < 1%) exhibited LIRA‐scores above the threshold (LIRA‐score C = 12.434, LIRA‐score D = 13.043). Further, we analyzed the expression of model genes in the four patients (Figure 4C). Genes associated with poor prognosis (“advanced”), such as MAFK and FAT1, were overexpressed in patients with low LIRA‐score. In contrast, genes associated with good prognosis (“favorable”), such as IDO1 and CD96, were highly expressed in patients with high LIRA‐score. As expected, patients with low LIRA‐score had an unfavorable prognosis, whereas those with high LIRA‐score consistently achieved better PFS. CT imaging further underscored the higher predictive ability of LIRA‐score (Figure 4D). Notably, LIRA successfully identified patients with low or negative PD‐L1 expression (TPS < 1%) who nonetheless responded to ICIs, as well as patients with high PD‐L1 expression who failed to benefit from treatment.

These results demonstrate that, by leveraging a transcriptomic reference database, LIRA can provide accurate immunotherapy benefit predictions for single samples and small independent cohorts. These findings support its clinical utility for personalized prognosis in advanced NSCLC.

### 2.6 A Low LIRA‐Score Suggests Acetylation Activation and an Immunosuppressive Microenvironment

To explore the underlying mechanisms contributing to the predictive capacity of LIRA in advanced NSCLC patients treated with ICIs, we delved into the transcriptomic data from the OAK and POPLAR cohorts. We first characterized the biological features of the 50 model genes (Figure S1, Supporting Information). A VIMP plot identified *SMOC1* as the top‐ranked contributor to the model; this gene has been previously associated with metastatic potential in pancreatic neuroendocrine tumors.^[^ ^31^ ^]^ Genes such as FAT1 and MAFK, which are associated with poor tumor prognosis,^[^ ^32^ ^]^ were also highly expressed in the low LIRA‐score group. Conversely, genes, such as IDO1 (a key enzyme in tryptophan catabolism) and CD96,^[^ ^33^, ^34^ ^]^ enriched in the high LIRA‐score group were largely involved in immune regulation and leukocyte functions. These data suggest that LIRA incorporates both protective and risk‐associated genes, allowing refined patient stratification.

**Figure 5:**
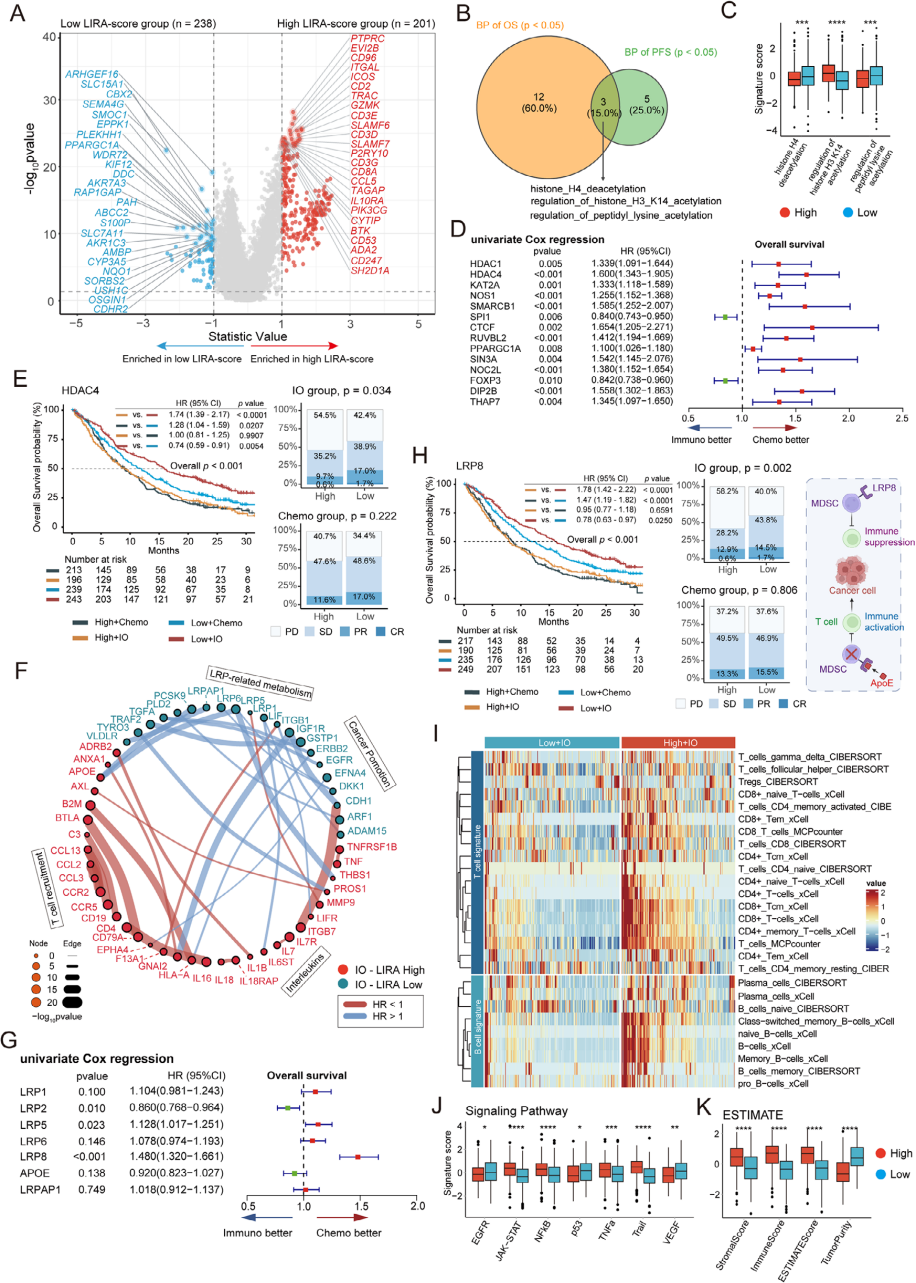
LIRA‐score is correlated with distinct transcriptomic and metabolic characteristics. A) Volcano plot depicting differentially expressed genes (*p* < 0.05; Statistic Value > 1) between low and high LIRA‐score groups from OAK and POPLAR with atezolizumab treatment (*n* = 439 patients). The top 25 genes overexpressed in the low and high LIRA‐score groups are shown. B) Venn diagram highlighting acetylation‐ and deacetylation‐related gene signatures that show significant associations in univariate Cox analyses for overall survival (OS, orange) and progression‐free survival (PFS, green) (*p* < 0.05). These pathway scores, derived from GO‐BP terms and computed using ssGSEA, were analyzed in patients receiving atezolizumab. C) Boxplots displaying normalized pathway enrichment scores of acetylation‐ and deacetylation‐related signatures that demonstrated statistically significant associations in both OS and PFS between high and low LIRA‐score groups treated with atezolizumab. D) Forest plot showing the HRs, 95% CIs, and *p*‐values of acetylation and deacetylation related genes based on univariate Cox analysis for OS in patients receiving atezolizumab. E) Kaplan–Meier curves (left) comparing OS among low and high HDAC4 expression groups in the OAK‐POPLAR combined cohort. The bar plots display BOR rates between low‐ and high‐HDAC4 expression groups treated with atezolizumab (top‐right) and docetaxel (bottom‐right). F) Differential ligand–receptor interaction network between high and low LIRA‐score group. Different circles represent different genes colored according to the groups. Node size represents the statistical significance of each gene. Edges are weighted by the statistical significance of ligand–receptor interactions between high and low score group. G) Forest plot showing the HRs, 95% CIs, and *p* values of LRP family and related genes based on univariate Cox analysis for OS in patients receiving atezolizumab. H) Kaplan–Meier curves (left) comparing OS among low and high LRP8 expression groups in the OAK‐POPLAR combined cohort. The bar plots show the BOR rates between low‐ and high‐LRP8 expression groups treated with atezolizumab (top‐middle) and docetaxel (bottom‐middle). The mechanistic diagram illustrates LXR/ApoE signaling modulates MDSC survival through the LRP8 receptor, thereby regulating the antitumor activity of T cells (adapted from^[^ ^42^ ^]^ using BioRender). I) Heatmap showing TME‐infiltrating cell signature score (red = high expression; blue = low expression) of each patient in high (red) and low (blue) score groups. Rows of the heatmap show expression of TME‐infiltrating cell signatures (z‐score) calculated by CIBERSORT, MCP‐counter, and xCell. J) Boxplots showing normalized pathway enrichment scores in high and low LIRA‐score groups with atezolizumab treatment. K) Boxplots showing normalized ESTIMATE score in low and high LIRA‐score groups with atezolizumab treatment. P value in (C) (J) (K) was calculated using a two‐sided Mann–Whitney U test. *p < 0.05, **p < 0.01, ***p < 0.001, ****p < 0.0001. ns, not significant compared to the corresponding group. Center line, box limits, and whiskers represent the median, interquartile range, and 1.5× interquartile range, respectively. HR, hazard ratio; CIs, confidence intervals; FC, fold change.

We next performed differential expression analysis (*p* < 0.01, Wilcoxon test) between the high and low LIRA‐score groups in patients treated with ICIs (*n* = 439) (**Figure** **5**A; Figure S7A and Table S6, Supporting Information). Pathway enrichment and gene signatures were comprehensively explored (Figure S7B–D, Supporting Information). Gene Ontology (GO) analysis^[^ ^35^ ^]^ revealed that genes upregulated in the high LIRA‐score group were enriched in immune‐activating processes and lymphocyte‐related functions, suggesting a more favorable immune phenotype in this group. In contrast, the genes overexpressed in the low LIRA‐score group were enriched in the acetylation‐related pathways (Figure S7B, Supporting Information). These findings were further validated using gene set enrichment analysis (GSEA)^[^ ^36^ ^]^ based on the GO and MSigDB HALLMARK^[^ ^37^ ^]^ databases (Figure S7C,D, Supporting Information).

To investigate potential mechanisms of immunotherapy resistance in low LIRA‐score patients, we focused on acetylation and deacetylation processes. A total of 49 gene sets related to histone acetylation were curated from the GO‐BP database and scored using single‐sample GSEA (ssGSEA). Univariate Cox regression identified three pathways significantly associated with both PFS and OS: histone H4 deacetylation, regulation of histone H3K14 acetylation, and regulation of peptidyl‐lysine acetylation (Figure 5B,C). These results indicate a potential link between histone modification and immunotherapy resistance in the low LIRA‐score group. To identify specific genes that may influence the outcome of ICIs, we conducted univariate Cox regression and Kaplan–Meier analyses. HDAC4 emerged as a significant factor, with high expression levels associated with poor PFS and OS (Figure 5D). The patients with low HDAC4 expression receiving atezolizumab had better outcomes (Figure 5E; Figure S8A,B, Supporting Information). Clinical studies have shown that NSCLC patients treated with combined HDAC inhibitors (HDACi) and ICIs derive clinical benefit,^[^ ^38^, ^39^ ^]^ suggesting that HDAC4‐mediated deacetylation may contribute to resistance in low LIRA‐score patients.^[^ ^40^ ^]^

Next, we assessed ligand–receptor (LR) interactions across LIRA‐score groups (see Methods). LR interaction analyses indicated that the low LIRA‐score group had more malignant and LRP‐related metabolism interaction compared to the high LIRA‐score group (Figure 5F–H; Table S7, Supporting Information).^[^ ^41^ ^]^ In particular, the APOE_LRPAP1_LRP8 interaction was significantly correlated with both poor PFS and OS (Figure S8C–E, Supporting Information). Expression analysis confirmed that LRP family genes were upregulated in the low LIRA‐score group (Figure S8F, Supporting Information), with LRP8 showing the strongest association with poor outcomes (HR for OS = 1.480, 95% CI: 1.320–1.661, *p* < 0.001; HR for PFS = 1.286, 95% CI: 1.163–1.423, *p* < 0.001; Figure 5G,H; Figure S8G, Supporting Information). Previous studies have shown that LRP8–APOE signaling contributes to the formation of an immunosuppressive TME,^[^ ^42^, ^43^, ^44^ ^]^ implicating LRP8 as a potential mediator of ICI resistance in low LIRA‐score group. Consistently, across multiple NSCLC cohorts, both LRP8 and HDAC4 were found to be highly expressed in patients with low LIRA‐scores. (Figure S8H–K, Supporting Information).

To explore the immune microenvironment characteristics underlying LIRA stratification, we analyzed immune cell infiltration, tumor‐intrinsic signaling, and tumor purity across groups. The high LIRA‐score group exhibited increased infiltration of T and B cells and activation of antitumor immune pathways such as JAK–STAT and NF‐κB (Figure 5J), consistent with a favorable response to ICI therapy.^[^ ^45^, ^46^ ^]^ In contrast, the low LIRA‐score group showed higher tumor purity and significantly reduced levels of TILs and overall immune cell infiltration (Figure 5I,K).

Taken together, these data suggest that high LIRA‐scores are associated with increased immune cell infiltration and activation of antitumor immune pathways. In contrast, immunotherapy resistance in the low LIRA‐score group may be driven by immunosuppressive factors such as LRP8 signaling and histone deacetylation pathways.

### 2.7 Immune Profiling of Tumors with Different LIRA‐Scores Reveals Distinct Cellular Composition and Interactions

**Figure 6:**
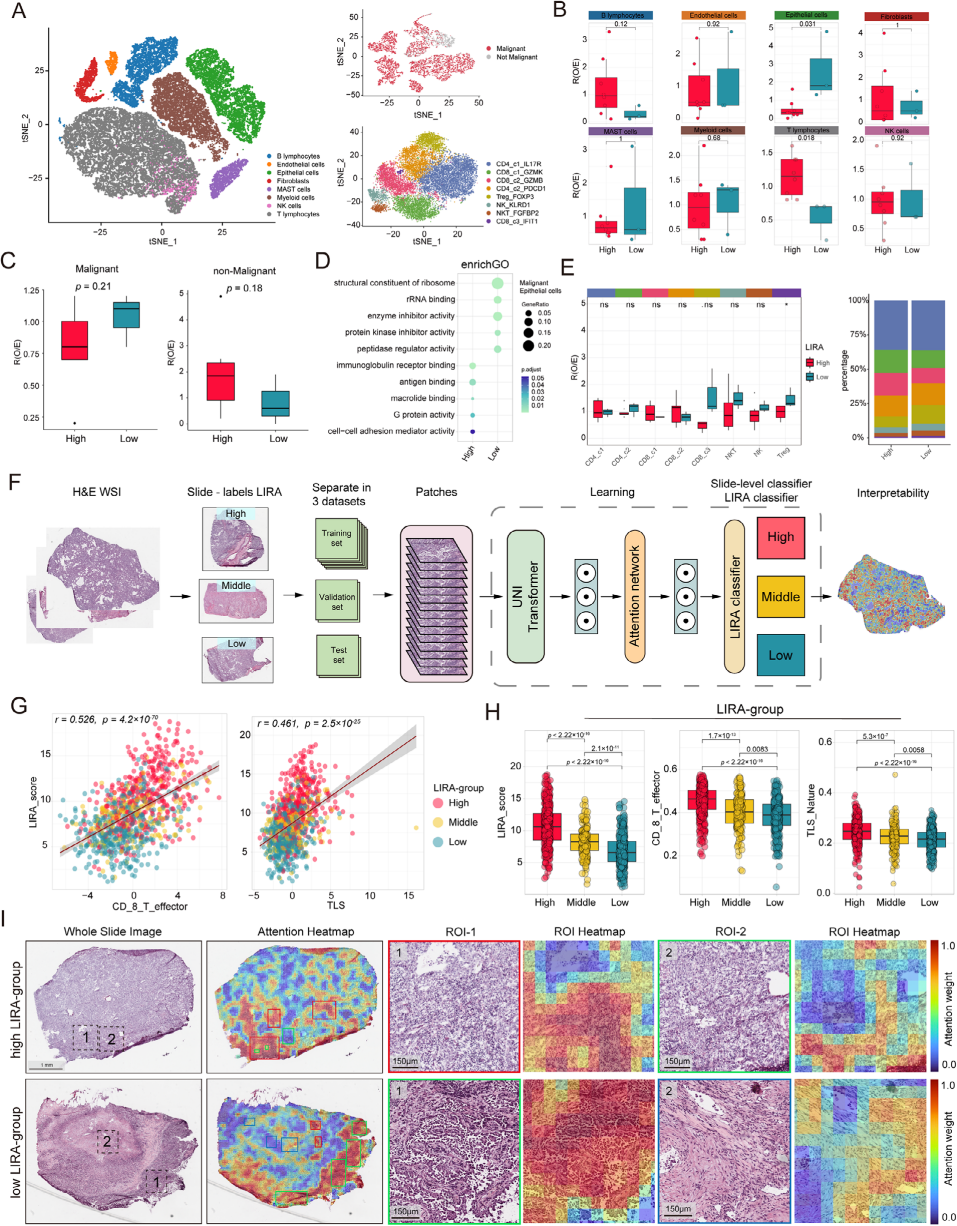
Immune profiles of tumors with high LIRA‐score group versus low LIRA‐score group. A) t‐SNE plots show 41779 single cells from tumor tissue from 11 NSCLC patients. Points are color‐coded by major cell types (left), epithelial cell subsets classified by malignancy status (top right), and T cell subsets (bottom right). B) Boxplots comparing the proportion of eight major cell types among total cells between patients with high and low LIRA‐score groups. C) Boxplots comparing the proportion of malignant and non‐malignant epithelial cells between high and low LIRA‐score groups. D) Bubble plots displaying the results of enrichment analysis of epithelial cells in the high and low LIRA‐score groups. E) Boxplots and percentage bar plots comparing the proportion of T cell subtypes between high and low LIRA‐score groups. F) Overview of the training workflow for the LIRA‐classifier. After patient grouping based on LIRA‐score and WSI segmentation (left), tissue patches are extracted. Each patch is encoded into a 1024‐dimensional feature vector using the UNI transformer. During training and inference, the encoded features are fed into a CLAM framework (center), where an attention network aggregates patch‐level information into a slide‐level representation to generate both classification predictions and attention heatmaps (right). G) The correlation plot showing the Spearman correlation between the LIRA‐score and the signature score of CD8+ T effector and TLS. H) Boxplots displaying LIRA‐score, signature score of CD8 T effector and TLS in the LIRA‐groups identified by LIRA‐classifier: High (high LIRA‐group, red), Middle (middle LIRA‐group, yellow), and Low (low LIRA‐group, blue). I) Whole‐slide attention heatmaps for each slide were generated by calculating the attention scores for the predicted class of the model across patches tiled with a spatial overlap of 25%. The representative slides from high and low LIRA‐group tumors were annotated by two pathologists, who identified the cell types on the WSI: lymphocyte regions (red boxes), tumor regions (green boxes), and fibroblast regions (blue boxes). The ROI (black boxes 1 and 2) and their corresponding ROI‐heatmaps are shown at 20× magnification. P values in (B) and (C) were calculated using a two‐sided Wilcoxon test, and in (H) using a two‐sided Mann–Whitney U test. *p < 0.05, **p < 0.01, ***p < 0.001, ****p < 0.0001; ns indicates not significant compared to the corresponding group. The center line, box limits, and whiskers represent the median, interquartile range, and 1.5× the interquartile range, respectively. Epi, epithelial cells; TLS, Tertiary lymphoid structures; ROI, Regions of interest.

Using bulk RNA‐seq data, we initially explored the factors that may contribute to differential immunotherapy responses in tumors with varying LIRA‐scores. However, TME analysis based on bulk RNA‐seq data provides only a relatively coarse view of cellular composition and intercellular interactions. To better define the immune profiles of tumors with different LIRA‐scores, we analyzed a scRNA‐seq dataset consisting of 41779 cells collected from 11 NSCLC patients (Table S8, Supporting Information).^[^ ^47^ ^]^ A pseudo‐bulk approach was applied to calculate the LIRA‐score for each sample, and patients were divided into high (*n* = 8) and low (*n* = 3) LIRA‐score groups based on the cohort mean. Using graph‐based clustering and t‐distributed stochastic neighbor embedding (t‐SNE), we identified eight major cell types, including epithelial (alveolar and cancer cells), stromal (fibroblasts and endothelial cells), and immune cells (T, NK, B, myeloid, and mast cells) (**Figure** **6**A; Figure S9A–C, Supporting Information). We observed an increased number of T cells in the high LIRA‐score group, while the low LIRA‐score group had a higher proportion of epithelial cells (Figure 6B). Further graph‐based clustering identified six T cell subtypes. The InferCNV R package was used to detect initial copy number variations (CNV) per region in epithelial cells.^[^ ^48^ ^]^ Malignant epithelial cells were distinguished from non‐malignant epithelial cells based on perturbations in CNV signals (Figure 6A, **see Methods**). We observed an increased number of malignant cells in the low LIRA‐score group, while non‐malignant cells were more prominent in the high LIRA‐score group. Although these differences were not statistically significant, they showed a trend. (Figure 6C). Pathway enrichment analyses showed strong enrichment of immunoglobulin receptor binding and antigen binding pathways in epithelial cells from the high LIRA‐score group (Figure 6D; Figure S9D,E, Supporting Information). Notably, the proportion of Treg cells was higher in the low LIRA‐score group (Figure 6E). Cellular communication analysis revealed that T cells secreted CCL, which acted on myeloid cells and epithelial cells, while fibroblasts secreted CXCL, which acted on B cells and T cells (Figure S10A–D, Supporting Information).

### 2.8 LIRA‐Score Prediction Using Deep Learning on NSCLC WSI Data

To further elucidate the reasons for different immunotherapy outcomes between patients with different LIRA‐scores, we utilized WSI data from TCGA‐LUAD and TCGA‐LUSC to train a pathology deep learning model based on the Cluster‐based Attention Multiple Instance Learning framework, termed the LIRA‐classifier (Figure 6F).^[^ ^49^ ^]^ The model can predict a patient's LIRA‐score category (LIRA‐group) from WSIs and generate attention heatmaps to visualize the relative importance of each region within the WSI to the model's prediction.

Using the LIRA‐classifier, patients from the TCGA‐LUAD and TCGA‐LUSC cohorts were stratified into high, medium, and low LIRA‐groups (Table S9, Supporting Information). Transcriptomic analysis showed a strong correlation between LIRA‐score and the signature scores of CD8+ T effector cells and tertiary lymphoid structures (TLS) (Figure 6G). We further explored whether the LIRA‐groups predicted by the LIRA‐classifier shared similar characteristics with LIRA‐score. The results revealed a progressive decrease in LIRA‐score, CD8+ T cell score, and TLS score from the high group to the low group, supporting the predictive accuracy of the LIRA‐classifier (Figure 6H).

Next, we analyzed the high‐attention regions identified by the model to determine pathological features potentially contributing to LIRA‐score predictions (Figure 6I). Two pathologists annotated the cell types and morphologies within these high‐attention regions. In the high LIRA‐group, high‐attention regions were predominantly enriched with lymphocytes, while in the low LIRA‐group, these regions were mainly composed of tumor cells and fibroblasts (Figure 6I). It was reported that interactions between tumor cells and fibroblasts could inhibit T cell infiltration and contribute to immunotherapy resistance.^[^ ^16^ ^]^ These observations are consistent with the transcriptomic findings. These findings indicate that the presence of lymphocytes contributes to the better immunotherapy outcomes observed in patients with high LIRA‐score, whereas the enrichment of tumor cells and fibroblasts is associated with poor prognosis in patients with low LIRA‐score.

Together, these results provide a pathological basis for the predictive power of the LIRA‐score and suggest that the deep learning model may enable rapid prediction of immunotherapy response in NSCLC patients using WSIs.

## 3 Discussion

In the multicenter study, we collected extensive transcriptomic data from 891 advanced NSCLC patients treated with either atezolizumab monotherapy or docetaxel in the OAK and POPLAR trials. Based on these data, we developed a predictive model, LIRA, using the random survival forest method to predict the survival benefit from ICIs in advanced NSCLC.^[^ ^26^, ^27^ ^]^ Through validation across multiple cohorts, LIRA could accurately predict clinical benefit for patients with ICI treatment, independent of other clinical variables or molecular biomarkers. Additionally, we found that LIRA can identify patients with PD‐L1‐negative and ‐low expression who could benefit from ICI therapy.

Currently, the treatment of advanced NSCLC relies on molecular testing for predictive biomarkers, including oncogenic drivers, to determine eligibility for targeted therapies such as those for *EGFR, ALK, ROS1, BRAF, HER2, MET, RET*, or *KRASG12C*. TMB and PD‐L1 expression levels on tumor cells guide decisions for ICI‐based therapies.^[^ ^28^ ^]^ In the absence of driver mutations, alternative treatment strategies are needed.^[^ ^50^, ^51^, ^52^ ^]^ The introduction of ICIs has altered the treatment landscape, offering durable responses for a subset of patients without driver alterations. However, several phase III trials reported that only 15–25% of patients with advanced NSCLC respond to ICI treatment, highlighting the need for better predictive biomarkers to identify responders and optimize clinical decision‐making.^[^ ^53^, ^54^ ^]^ Previous studies have reported that genetic alterations such as *EGFR* mutations and ALK rearrangements are negatively associated with ICI efficacy.^[^ ^4^, ^24^ ^]^ In the randomized, open‐label, international phase III study CheckMate057, the *KRAS*‐mutant NSCLC subgroup showed a significant OS benefit from nivolumab monotherapy than docetaxel (HR: 0.52, 95% CI: 0.29 – 0.95), suggesting that driver mutation status may help inform immunotherapy decisions.^[^ ^55^ ^]^ However, significant inter‐patient heterogeneity exists within genomic subgroups, making it difficult to predict immunotherapy outcomes based solely on driver mutations.

Clinical studies have demonstrated that favorable ICI treatment responses in NSCLC are related to the level of PD‐L1 expression and TMB.^[^ ^1^, ^2^, ^56^ ^]^ Therefore, PD‐L1 expression and TMB have been widely used to predict the treatment response of ICI therapy in clinical practice.^[^ ^6^ ^]^ However, it was reported that a subset of NSCLC patients with low or negative PD‐L1 expression can exhibit durable responses to ICI treatment.^[^ ^13^ ^]^ Thus, there is a pressing need to identify novel and more specific biomarkers to predict which patients could gain a survival benefit through ICI treatment.

T‐cell‐derived biomarkers, such as the IFN‐γ RNA signature and T‐cell‐inflamed GEP, also serve as predictive biomarkers for ICI treatment in clinical trials.^[^ ^14^ ^]^ While GEP has been shown to predict immunotherapy benefits in pan‐cancer patients, including head and neck squamous cell carcinoma and melanoma, its clinical relevance in NSCLC has not been thoroughly investigated.^[^ ^12^, ^57^ ^]^ The advent of AI and machine learning algorithms offers a novel approach for comprehensive profiling of a patient's TME and immunotherapy prediction.^[^ ^18^ ^]^ Previously, several studies investigated the application of a radiomics‐based model to predict the survival benefit of ICIs.^[^ ^19^ ^]^ Tang *et al*. constructed predictive models using machine learning methods, including Decision Trees, Boosted Trees, Random Forests, and Support Vector Machines based on image features from 422 patients. Random Forest achieved the best predictive performance of AUC = 0.938 among the models. The comparative study substantiated the value of machine learning methods in predicting benefits from immunotherapy.^[^ ^58^ ^]^ Beyond radiomics, previous research has demonstrated the feasibility of developing machine learning predictive models based on transcriptomes.^[^ ^59^ ^]^ However, there are still fewer transcriptome‐based machine learning models capable of accurately predicting responses of patients with NSCLC to immunotherapy.^[^ ^22^ ^]^ Therefore, we established a transcriptome‐based assessment model through a machine learning method to re‐evaluate the value of transcriptomics in predicting immunotherapy outcomes. During feature engineering, three selection criteria were applied to identify genes relevant to immunotherapy benefit: 1) statistical significance in univariate Cox proportional hazards regression (*p* < 0.01, HR < 1); 2) significant interaction with treatment regimen (atezolizumab versus docetaxel), identified using the “subgroupAnalysis” function of Publish R package (*p* for interaction < 0.05); and 3) top 50 genes ranked by VIMP in the random survival forest model. By inputting bulk RNA‐seq data, LIRA calculates a patient‐specific LIRA‐score to predict their likelihood of responding to ICIs.

The results indicated that LIRA could identify patients who could benefit from ICI treatment. In the training and internal validation cohorts of second‐ and third‐line PD‐L1 blockade monotherapy or docetaxel, high LIRA‐score group treated with atezolizumab achieved longer PFS and OS compared to those treated with docetaxel. In contrast, patients in the low LIRA‐score group who received atezolizumab experienced earlier tumor progression than those receiving docetaxel. We further validated the predictive performance of LIRA in external cohorts. The results showed that a high LIRA‐score was associated with significantly better survival benefits in patients treated with PD‐1 blockade combined with chemotherapy or anti‐angiogenic therapy in two independent, external cohorts (ORIENT‐11 and in‐house NFH cohort).^[^ ^25^ ^]^ We found that the survival benefit was similar whether patients with low LIRA‐score received chemotherapy or ICIs plus chemotherapy in the ORIENT‐11 cohort. In non‐ICI‐treated NSCLC cohorts and ICI‐treated cohorts from other cancer types, LIRA demonstrated only limited predictive performance, indicating its specificity for NSCLC patients receiving ICIs. When compared with clinical biomarkers, LIRA outperformed both TMB and TPS in predicting immunotherapy outcomes. Importantly, LIRA's predictive ability was not affected by *EGFR*, *STK11*, or *KEAP1* mutation status.

Collectively, these findings suggest that LIRA provides superior predictive power compared to existing biomarkers. It not only predicts survival benefit in patients receiving PD‐L1 blockade monotherapy but also effectively identifies responders among those treated with PD‐1 blockade combined with chemotherapy. Furthermore, this analysis showed that patients with low LIRA‐score treated with ICI monotherapy were more likely to experience early disease progression than those receiving chemotherapy. Previously reported real‐world data showed that the survival benefit of combination ICI with chemotherapy was comparable to that of ICI monotherapy. However, ICI monotherapy was associated with higher rates of early progression.^[^ ^50^ ^]^ This finding is consistent with the results, indicating that chemotherapy may offer greater benefit than immunotherapy for patients with a low LIRA‐score.

To facilitate the clinical translation of LIRA, we constructed a reference database based on bulk RNA‐seq data of patients with atezolizumab from OAK and POPLAR cohorts. This reference database enabled the computation of LIRA‐score for individual samples or small patient cohorts, supporting single‐sample level immunotherapy prediction. Single‐patient analysis from the NFH cohort demonstrated that LIRA effectively identified NSCLC patients with low PD‐L1 expression who could benefit from ICI therapy, demonstrating its clinical translation value. Wi rapid development and widespread application of sequencing technologies, RNA‐seq has evolved into a mature and cost‐effective analytical technique.^[^ ^60^ ^]^ Although RNA‐seq requires slightly more time than PD‐L1 immunohistochemistry assays, its cost has been markedly reduced and is now even lower than that of PD‐L1 IHC assays. Furthermore, RNA‐seq is both faster and more cost‐effective than TMB testing. Thus, alongside PD‐L1 expression testing, it is feasible to incorporate RNA‐seq for LIRA‐score calculation to assist in therapeutic decision‐making. Because LIRA‐score computation is time‐efficient and computationally inexpensive, it holds promise as an independent biomarker to complement PD‐L1 in identifying patients who are more likely to benefit from ICI therapy. Moreover, bulk RNA‐seq serves as a versatile data source that, when combined with appropriate computational pipelines, enables multi‐dimensional characterization of tumors, including gene expression, TME composition, and splicing variation. These analyses provide a comprehensive framework for evaluating immunotherapy sensitivity and identifying potential resistance mechanisms.^[^ ^61^ ^]^ Currently, an observational study based on the LIRA is underway (NCT06232265) to further validate its predictive performance and support clinical implementation.

Recognizing the potential predictive value of LIRA, we further investigated the immune profiles and resistance mechanisms of NSCLC tumors stratified by LIRA‐score. Transcriptomic analysis revealed that tumors with high LIRA‐score exhibited significantly higher infiltration of CD8+ T cells, CD4+ T cells, and plasma cells. ESTIMATE analysis indicated that tumors with low LIRA‐score had higher tumor purity. Pathway enrichment analysis revealed that the low LIRA‐score group was enriched in acetylation‐related pathways^[^ ^38^, ^40^ ^]^ and LRP‐related metabolism,^[^ ^42^, ^43^ ^]^ typically associated with immunosuppression. Further analysis of LR interactions and signature genes identified that LRP8 and HDAC4 were closely associated with immunotherapy resistance in the low LIRA‐score group. These findings suggest that tumors with a high LIRA‐score may exhibit an immune‐supportive TME phenotype.^[^ ^62^ ^]^

To further characterize the TME features of tumors with different LIRA‐scores, we performed an in‐depth analysis using scRNA‐seq data. We found an increased number of T cells in the high LIRA‐score group, whereas the proportion of epithelial cells was significantly lower in the high LIRA‐score group than in the low LIRA‐score group. Sub‐clustering analysis revealed an increased proportion of Treg cells in tumors with low LIRA‐score. Additionally, based on perturbations in CNV signals, a higher number of malignant epithelial cells was identified in the low LIRA‐score group. Studies have shown that both Treg cells and malignant epithelial cells can suppress effective antitumor immunity through different mechanisms, partially explaining the observed resistance to ICIs in patients with low LIRA‐score.^[^ ^63^, ^64^ ^]^ Overall, these findings suggest that tumors with low LIRA‐score are characterized by an immunosuppressive phenotype.

These results provide a mechanistic rationale for the superior predictive performance of LIRA relative to TPS and TMB. Traditional biomarkers such as TPS and TMB offer limited predictive performance due to their single‐dimensional nature. TPS reflects PD‐L1 expression only on tumor cells, while TMB serves as an indirect proxy for tumor antigenicity.^[^ ^13^, ^65^, ^66^ ^]^ In contrast, LIRA was developed using 50 model genes derived from transcriptomic data through rigorous feature selection. These targeted genes enable LIRA to capture a comprehensive molecular snapshot of the tumor and its microenvironment, encompassing immune activation signals (e.g., CD8+ T cell infiltration, B cell infiltration), immunosuppressive features (e.g., Treg enrichment, histone modification, LRP8‐mediated immunometabolism), and tumor‐intrinsic characteristics. By integrating both intrinsic and extrinsic immune‐related signatures, LIRA provides enhanced stratification power across heterogeneous patient populations and therapeutic contexts.

AI‐based approaches have gained increasing attention in cancer research, particularly in digital pathology, where they show great potential in tumor classification, diagnosis, and therapeutic response prediction. In addition, explainable AI can generate new insights.^[^ ^67^ ^]^ Mehrdad *et al.* developed a deep learning−based stratification model for predicting ICI treatment outcomes from WSIs.^[^ ^68^ ^]^ Zhao *et al*. developed an AI‐based method to predict gene mutation in NSCLC patients.^[^ ^69^ ^]^ Other studies have also reported the use of deep learning for predicting immune and inflammatory gene signatures,^[^ ^70^ ^]^ collectively demonstrating the extensibility and translational potential of AI in oncology. Therefore, we applied a deep learning approach to understand the pathological differences between high and low LIRA‐score tumors and to enable rapid prediction of LIRA‐score categories (LIRA‐group) from WSIs. Using the CLAM framework,^[^ ^49^ ^]^ we developed a deep learning model, called LIRA‐classifier, to predict LIRA‐group from WSIs and generate interpretable attention heatmaps that show each region's contribution to model predictions. In high LIRA‐group samples, high‐attention regions were enriched in lymphocyte infiltration, whereas in low LIRA‐group samples, they predominantly consisted of tumor cells and fibroblasts. These observations help explain why patients with high LIRA‐score achieve better immunotherapy outcomes. This further supports the utility of LIRA as a predictive biomarker for NSCLC immunotherapy. Moreover, these results suggest that deep learning models based on WSI data can provide a rapid, non‐invasive strategy for immunotherapy response prediction.

There remain limitations to this study. First, the development of LIRA was based solely on bulk transcriptomic data. Although we validated it across multiple cohorts, the lack of integration with other omics data, such as single‐cell, genomic, or metabolomic data, potentially limits its specificity. Constructing a more accurate predictive model for patient immunotherapy response by incorporating multi‐omics data is a direction for future research. Second, the performance of LIRA may be influenced by sample and technical heterogeneity. Biological heterogeneity arises from tumor complexity, while technical variation may result from differences in sequencing platforms or sample processing methods (e.g., frozen tissue versus FFPE).^[^ ^60^, ^71^, ^72^ ^]^ Third, the retrospective nature of the training cohort introduces potential biases, which may affect the predictive accuracy of LIRA. Furthermore, we did not exclude patients with activating driver mutations in the model training, which may introduce certain variables into the prediction of the model. Additionally, many patients in both the training and validation cohorts lacked TPS and TMB data, which limited the statistical power for direct comparison between LIRA and existing biomarkers. This also precluded accurate assessment of a combined biomarker model incorporating LIRA, TPS, and TMB. Finally, while we performed initial microenvironmental analyses and identified key genes to interpret the predictive ability of LIRA, a deeper mechanistic investigation is essential to understand the biological basis of immunotherapy resistance in the low LIRA‐score group. We are actively pursuing identifying the primary regulatory mechanisms of the feature genes, including LRP8 and HDAC4, through both mechanistic and clinical validation.

## 4 Conclusion

In conclusion, we developed and validated LIRA using machine learning and transcriptomes to predict the survival benefit of ICI treatment in patients with advanced NSCLC. By incorporating gene‐level feature engineering based on survival outcomes and treatment interactions, LIRA achieved enhanced performance in prognostic stratification of patients treated with immunotherapy. LIRA exhibits superior predictive performance in patients receiving PD‐L1 blockade monotherapy and PD‐1 blockade combination therapy. Importantly, LIRA can identify patients likely to experience early disease progression with immunotherapy, suggesting that patients with low LIRA‐score may benefit more from chemotherapy than immunotherapy alone.^[^ ^50^ ^]^ This provides an essential reference for clinical decision‐making.

## 5 Experimental Section

### Patient Cohorts

The study collected clinical and transcriptomic data from multiple cohorts for model development and validation. The transcriptomic data of 891 pre‐treatment NSCLC tumors were obtained from the open‐label, randomized Phase II POPLAR (NCT01903993)^[^ ^23^ ^]^ and Phase III OAK (NCT02008227).^[^ ^24^ ^]^ These trials evaluated atezolizumab versus docetaxel in patients with NSCLC who progressed following first‐line platinum‐based chemotherapy. The 891 transcriptomes of OAK (*n* = 192) and POPLAR (*n* = 699) cohorts were randomly divided into a training cohort (*n* = 625) and an internal validation cohort (*n* = 266) at a ratio of 7:3.

The transcriptomic data of 171 pre‐treatment NSCLC tumors were from the multi‐center randomized Phase III ORIENT‐11 (NCT03607539),^[^ ^25^ ^]^ which evaluated patients treated with sintilimab or placebo in combination with pemetrexed and platinum. This cohort was used as an external validation cohort (ORIENT‐11 cohort).

Pre‐treatment tumor specimens from 65 patients with advanced NSCLC who received ICI monotherapy or ICI combination therapy in the in‐house NFH cohort between March 2017 and June 2021 were collected from Nanfang Hospital and Guangdong Provincial People's Hospital. This cohort was also used as an external validation cohort.

Transcriptomic data of 132 pre‐treatment SCLC tumors were obtained from the multicenter, randomized Phase III IMpower133 trial (NCT02763579), which evaluated atezolizumab in combination with etoposide in chemotherapy‐naive patients with extensive‐stage SCLC.^[^ ^73^ ^]^ Transcriptomic and corresponding clinical data of 348 urothelial carcinoma patients from the IMvigor210 trial (NCT02108652) were downloaded from the IMvigor210CoreBiologies R package (*n* = 348).^[^ ^74^ ^]^ IMpower133 and IMvigor210 cohorts were used to validate the model in non‐NSCLC immunotherapy settings.

Transcriptomes and clinical data of NSCLC patients with non‐ICI treatment were from TCGA‐LUAD, TCGA‐LUSC, GSE37745, GSE157011, GSE81089, GSE31210, GSE28509, and GSE131907. These cohorts were used to validate the model and further analysis in non‐immunotherapy NSCLC contexts. See Table 1 and Tables S1–S3 (Supporting Information) for more details.

The ORIENT‐11 clinical trial and NFH cohort study were approved by the institutional review boards and the ethics committees of Nanfang Hospital (NFEC‐2019‐265), respectively. All participants provided written informed consent prior to enrollment. All studies were conducted according to the Declaration of Helsinki. All patient‐relevant information was anonymized and de‐identified. The study, following the Standards for Reporting of Diagnostic Accuracy (STARD) guidelines, was completed. Ethics approval and patient‐informed consent for The Cancer Genome Atlas (TCGA), Gene Expression Omnibus (GEO), and European Genome‐phenome Archive (EGA) were waived due to their public availability.

### RNA Sequencing

Fresh frozen NSCLC tissues were obtained from 65 patients with advanced NSCLC. Total RNA was extracted from tumor tissue using Trizol and digested with DNase I. Then mRNA was purified from total RNA using oligo(dT)‐attached magnetic beads and subsequently fragmented. Following that, according to the manufacturer's protocol, sequencing libraries were generated using NEBNext Ultra RNA Library Prep Kit for Illumina (NEB, USA). The library preparations were sequenced on a DNBSEQ‐T7RS (MGI), generating 100 bp paired‐end reads.

The RNA sequencing data were processed through a series of steps to ensure high‐quality gene expression results. Initially, quality control (QC) checks were performed on the raw sequence data using fastp (v0.23.4).^[^ ^75^ ^]^ For the alignment of the trimmed reads, the STAR aligner (v.2.7.1a) was employed. Reads were mapped to the human reference genome GRCh38 (v111), utilizing the comprehensive gene annotations provided by the GENCODE project (release 44). STAR was configured to perform a two‐pass alignment. Post‐alignment, STAR generated gene‐level quantification in the form of read counts per gene. Finally, the read counts obtained from STAR were used to assemble a gene expression matrix.

### Development of a Transcriptome‐Based Machine Learning Assessment Model

This study performed feature engineering to identify genes associated with treatment outcomes and therapeutic regimens for use in the development of an immunotherapy response prediction model. Feature gene selection was performed in the training cohort following the steps (Figure 1). First, univariate Cox proportional‐hazards regression analysis was used to evaluate the relationship between gene expression and survival outcomes (OS and PFS). Genes with *p* < 0.01 were selected for further analysis (Table S4, Supporting Information). Second, subgroup analysis was performed to assess potential interaction effects between feature genes and treatment regimens (docetaxel and atezolizumab). Specifically, this analysis was based on Cox regression to evaluate interaction terms between individual genes and treatment effects. To enable prediction of PFS across treatment modalities in NSCLC patients, the univariate Cox regression between PFS and treatment served as the main regression model. The *p*‐value for interaction and HR were calculated to explore both the stability and interaction effect of feature genes using the “subgroupAnalysis” function of the Publish R package in the training cohort.^[^ ^76^ ^]^ The *p*‐value for interaction was obtained with a likelihood ratio test comparing the main regression analysis with the interaction model. The feature genes with *p* for interaction < 0.05 were retained. The *p*‐value for interaction was obtained through a likelihood ratio test comparing the main model with the interaction model. Finally, using the random survival forest algorithm, the top 50 genes ranked by VIMP were selected as model genes. Based on these, the predictive model, LIRA, was trained in the training cohort treated with atezolizumab to estimate survival probability in NSCLC patients according to the expression levels of the model genes (Table S4, Supporting Information). To evaluate the predictive performance of LIRA in combination with TMB and TPS, multimodal models via Cox regression were constructed and their performance was assessed accordingly.

The predictive accuracy of all models was evaluated using time‐dependent ROC analysis. The AUC was calculated and compared across the biomarkers using the comparison test. If the input gene expression matrix is missing any model genes, those genes will be omitted from LIRA‐score calculation.

### Construction of the Reference Database

To enable the assessment of LIRA‐score at the single‐sample level, RNA‐seq data were selected from NSCLC patients treated with atezolizumab in the OAK and POPLAR cohorts (*n* = 439) to construct a reference database. This reference database allows for the computation of LIRA‐score that are comparable across samples. The detailed procedure is as follows: After sequencing an individual sample, its count data were merged with the reference database. Batch effects were removed to generate batch‐corrected data. Next, the LIRA‐score was calculated based on the merged dataset. The optimal cutoff value for LIRA‐score was determined using the IOBR R package, based on PFS from the reference cohort.^[^ ^77^ ^]^ Samples with LIRA‐scores above this threshold were predicted to have favorable outcomes with ICI treatment, while those below the threshold were predicted to have poor responses.

### DEG and Functional Analysis

All differentially expressed gene analyses were conducted using IOBR R package.^[^ ^30^, ^77^ ^]^ The batch_wilcoxon function and Wilcoxon rank‐sum test were applied to identify differentially expressed genes between two groups based on log(TPM + 1). GO^[^ ^35^ ^]^ and GSEA^[^ ^36^ ^]^ were performed using the clusterProfiler R package.^[^ ^78^, ^79^ ^]^ By the approach of the GSEA algorithm, GO and MSigDB HALLMARK gene sets were used to estimate the pathway enrichment score.^[^ ^37^ ^]^ An adjusted *p* < 0.05 was considered the cutoff criterion.

### Estimation of LR Pairs and Pathway Enrichment Score

The EaSIeR R package was adopted to evaluate LR pairs based on gene expression patterns.^[^ ^41^ ^]^ The transcriptomic data were provided as input to EaSIeR, which derived group‐specific, system‐based signatures of the TME. A pairwise Wilcoxon test was used to identify different signatures and ligand–receptor interactions between groups.

### Inference of Cell Fraction and Calculating the Signature Score

Several computational tools (CIBERSORT,^[^ ^80^ ^]^ MCP‐counter,^[^ ^81^ ^]^ ESTIMATE,^[^ ^82^ ^]^ and xCell^[^ ^83^ ^]^) were integrated to estimate the infiltration of multiple cell types in the datasets mentioned above.^[^ ^30^ ^]^ Other prevalent gene signature scores concerning the TME, the tumor‐intrinsic pathways, and metabolism were calculated for each sample using the IOBR R package (https://iobr.github.io/book/) (Table S5, Supporting Information).^[^ ^30^ ^]^

### Quality Control of scRNA‐Seq Data

Expression matrices of scRNA‐seq of NSCLC patients were converted into a Seurat object using Seurat (v.4.3.0).^[^ ^47^, ^84^ ^]^ The cell QC based on deviations from median absolute deviations (MADs) is performed on the following thresholds: log10_total_counts >5 or <5 MADs, log10_total_features > or <5 MADs, pct_counts_in_top_20_features > or <5 MADs, and pct_counts_Mt >0.2.^[^ ^85^ ^]^ Subsequently, DecontX was used to distinguish cells from empty droplets containing only ambient RNA, and scDblFinder to eliminate potential doublets.^[^ ^86^, ^87^ ^]^ Next, SCTransform was applied to normalize, scale, and identify variable features of the data.^[^ ^88^ ^]^

### Dimensionality Reduction and Clustering

Twenty principal components were selected at a resolution of 0.5, and the FindNeighbors and FindClusters functions in the Seurat package were used for cell clustering. Cells were visualized using the Barnes‐Hut t‐SNE. The raw cell identity information was used in the data for the annotation of the main cell type. Subsequently, epithelial cells and T lymphocytes were divided into subsets for normalization, dimensionality reduction, and further clustering into subclusters using a similar strategy, thus allowing for the detection of heterogeneity within each cell type. The second round of clustering procedures for these cell types was the same as above.

### LIRA‐Score Grouping Using scRNAseq Data

Pseudobulk analysis was applied to estimate gene expression in the integrated Seurat object using the function AggregateExpression of the Seurat R package. Following gene expression normalization, the LIRA model was adopted to predict the LIRA phenotype of each sample.

### Relative Fold Change in Cell Clusters

The ratio (R) of observed (O) to random expected (E) cell number could adjust cell sampling bias for each patient, indicating cluster enrichment in a particular sample.^[^ ^89^ ^]^ The R(O/E) for each cluster in distinct samples was calculated using the Chi‐square test. The value of R(O/E) > 1 indicated the enrichment of the cell cluster in the sample. Differential abundance analysis for cell subclusters was performed by Wilcoxon tests between high LIRA‐score and low LIRA‐score samples.

### Copy Number Variants Analysis for Cancer Cells

The InferCNV R package (v1.10.1) with default parameters was used to detect initial CNVs per region in epithelial cells and to identify real cancer cells.^[^ ^48^ ^]^ The CNV signal was summarized with two parameters, including the mean squares of estimates across all windows in the *x*‐axis and the correlation of the CNV of each cell with the average of the top 5% of cells on the *y*‐axis.^[^ ^48^ ^]^ Epithelial cells showing perturbation in their CNV signal (>0.001 mean squares or >0.2 CNV correlation) were classified as malignant.

### Cellular Communication of scRNA‐Seq Data

The cell–cell interaction network in epithelial cells, NK cells, T lymphocytes, myeloid cells, B cells, endothelial cells, and fibroblasts in high LIRA‐score and low LIRA‐score samples was predicted using CellChat package (v1.6.0).^[^ ^90^ ^]^ LR pairs enriched between clusters were shown in high LIRA‐score and low LIRA‐score samples. Besides, Nichenetr package (v2.1.5) was also used to predict LR interactions.^[^ ^91^ ^]^ In this study, the receiver cell population was the “T‐lymphocytes” population, whereas the sender cell populations were all the main cell types. The gene sets of interest were genes that were differentially expressed in T‐lymphocytes in high LIRA‐score compared to low LIRA‐score samples.

### WSI Dataset

The WSI dataset used in this study consists of NSCLC samples from the TCGA‐LUAD (492 patients, 791 pathology slides) and TCGA‐LUSC (479 patients, 733 pathology slides) datasets. Non‐tumor adjacent slices, slides lacking corresponding diagnostic data, and damaged pathology slides were excluded from the analysis. On average, 2386 patches were extracted per slide at 10x magnification. The dataset was partitioned into training, validation, and testing (7:1:2) sets.

### WSI Preprocessing and Feature Extraction

The WSI was loaded into memory at a reduced resolution (4× downscaling) and converted from the RGB to HSV color space. A binary mask representing the tissue regions (foreground) is created by applying a threshold to the saturation channel after median blurring. This is followed by morphological closing to fill in small gaps and holes. The detected foreground objects' contours are then filtered based on an area threshold and stored for subsequent processing. After segmentation, the algorithm crops 256 × 256 patches from within the segmented tissue regions at 10× magnification.

After patching, the UNI model,^[^ ^92^ ^]^ a foundation model, was utilized, employing transfer learning techniques to extract features from each patch. Each patch is transformed into a 1024‐dimensional feature vector. The feature extraction process is conducted on two NVIDIA A5000 GPUs.

### Model Training and Validation for LIRA‐Classifier

The LIRA‐score was first calculated for the TCGA dataset, and the patients were categorized into three groups: high, middle, and low. Subsequently, a multi‐class classification model based on the CLAM framework, called LIRA‐classifier, was trained for LIRA‐score grouping.^[^ ^49^ ^]^ Training was performed using ten‐fold cross‐validation to ensure robust model evaluation. The model was configured with a dropout rate of 0.25. The learning rate was set at 0.0002 and optimized using the Adam optimizer. For loss functions, cross‐entropy loss was employed for slide‐level classification, coupled with SVM loss for instance‐level clustering. To address class imbalance, weighted sampling was enabled.

Training was conducted for a maximum of 200 epochs, with early stopping employed to halt the process when performance on the validation set plateaued. During each epoch, the model processed 1024‐dimensional feature vectors, applied attention mechanisms to select key instances, and updated its parameters to minimize the combined loss. After training, the performance of the model was evaluated on the test set for each fold, with metrics such as accuracy and AUC recorded. The results across all folds were averaged to provide an overall assessment.

### Interpretation of Model Predictions via Attention Heatmaps

To evaluate the significance of different regions within a slide in influencing the model's predictions, unnormalized attention scores were calculated for all patches extracted from the slide and then converted into percentile values, calibrated against the unnormalized scores across the entire slide. These scores were scaled between 0 (least attended) and 1 (most attended) and mapped to RGB colors using a diverging color scheme, with red regions indicating high attention (significant contribution to the model's decision) and blue regions indicating low attention. The resulting heatmaps were then overlaid onto the original WSIs with a transparency of 0.5, allowing for the simultaneous visualization of the heatmap and the underlying tissue structures.

### Statistical Analysis

The clinical benefit assessment for patients was based on RECIST (v1.1). In the single‐sample validation, DCB was defined as complete (CR)/partial response (PR) or stable disease (SD) that lasted > 6 months. NDB was defined as progressive disease (PD) or SD that lasted ≤ 6 months.^[^ ^93^ ^]^ PFS was calculated from the first treatment until PD, death, or loss of follow‐up. OS was calculated from the first treatment until death or loss of follow‐up.

The Shapiro–Wilk normality test was used to test the normality of the variables. For normally distributed variables, comparisons between two groups were performed using an unpaired Student's *t*‐test. For non‐normally distributed variables, the Mann–Whitney U test (Wilcoxon Rank Sum test) was applied. For comparisons of more than two groups, the Kruskal–Wallis and one‐way analysis of variance tests were used for non‐parametric and parametric methods, respectively. The correlation coefficient was computed by the Spearman and distance correlation analysis. The chi‐square and two‐sided Fisher's exact tests were used to analyze the contingency tables. The Kaplan–Meier method was used to generate survival curves for the subgroups in each dataset, and the log‐rank (Mantel–Cox) test was used to determine if they were statistically significant. All statistical analyses were conducted using R (version 4.2.3) (https://www.r‐project.org/), and the *p*‐values were two‐sided. *p*‐values less than 0.05 were considered statistically significant. The adjusted *P* value for multiple testing was calculated using the Benjamini–Hochberg correction.^[^ ^94^ ^]^

## Conflict of Interest

The authors declare no conflict of interest.

## Author Contributions

Z.W., Y.F., and X.H. are the co‐first authors. D.Z., Z.W., and Y.F. contributed to the conceptualization and study design. Z.W., Y.F., X.H., and G.M. contributed to collecting participant samples and clinical information. Z.W., Y.F., X.H., Q.M., X.L., and G.R. contributed to data analysis and interpretation with supervision from W.L., G.C., and D.Z. Y.F., D.Z., Z.W., and Y.Y. contributed to drafting the initial version of the manuscript and reviewing the manuscript. All the authors have read, discussed, and approved the final version of the manuscript. The corresponding author had full access to the data in the study and took responsibility for the integrity of the data and the accuracy of the data analysis.

## Supporting information



Supporting Information

Supplemental Table 1

## Data Availability

The raw data of transcriptome of the NFH cohort reported in this article has been deposited in the Genome Sequence Archive (https://ngdc.cncb.ac.cn/gsa/) under the accession number HRA003748. Access to the data requires approval from the corresponding authors. The clinical data of the OAK^[^ ^24^ ^]^ and POPLAR^[^ ^23^ ^]^ cohorts were available in the EGA database under accession codes EGAD00001008549 (OAK) and EGAD00001008548 (POPLAR).^[^ ^27^ ^]^ The bulk RNA‐seq data of OAK and POPLAR cohorts are available in the EGA database under accession codes EGAD00001007703. Additional clinical data of the OAK and POPLAR cohorts are available upon request from vivli.org. The bulk RNA‐seq and clinical data of the ORIENT‐11 cohort presented in this study are available from the lead contact upon reasonable request.^[^ ^25^ ^]^ The RNA‐seq and clinical data of the IMvigor210 cohort are available at IMvigor210CoreBiologies R package (EGAS00001002556).^[^ ^74^ ^]^ Raw and processed transcriptomic data and limited clinical data of IMpower133 are available in the EGA database under accession codes EGAS50000000138.^[^ ^73^ ^]^ The gene expression counts, WSIs and clinical data of TCGA‐LUAD and TCGA‐LUSC were downloaded from TCGA (https://portal.gdc.cancer.gov/). Publicly available gene expression datasets for NSCLC in the GEO database were downloaded from https://www.ncbi.nlm.nih.gov/geo/. Three normalized microarray datasets (GSE37745, GSE157011, and GSE31210), two RNA‐seq datasets (GSE81089 and GSE28509), and one scRNA‐seq dataset (GSE131907) were identified. The LIRA R package is available at https://github.com/LiaoWJLab/LIRA. Any additional information required to reanalyze the data reported in this paper is available from the lead contact upon request.
