# Supplementary material for: Predicting Immunotherapy Outcomes in NSCLC Using RNA and Pathology from Multicenter Clinical Trials
Source: Adv Sci (Weinh). 2025 Oct 29;13(3):e02037. doi: 10.1002/advs.202502037 (PMC12806205; doi:10.1002/advs.202502037)
Supplement: Supplementary file 1 — Supporting Information [file ADVS-13-e02037-s001.docx]

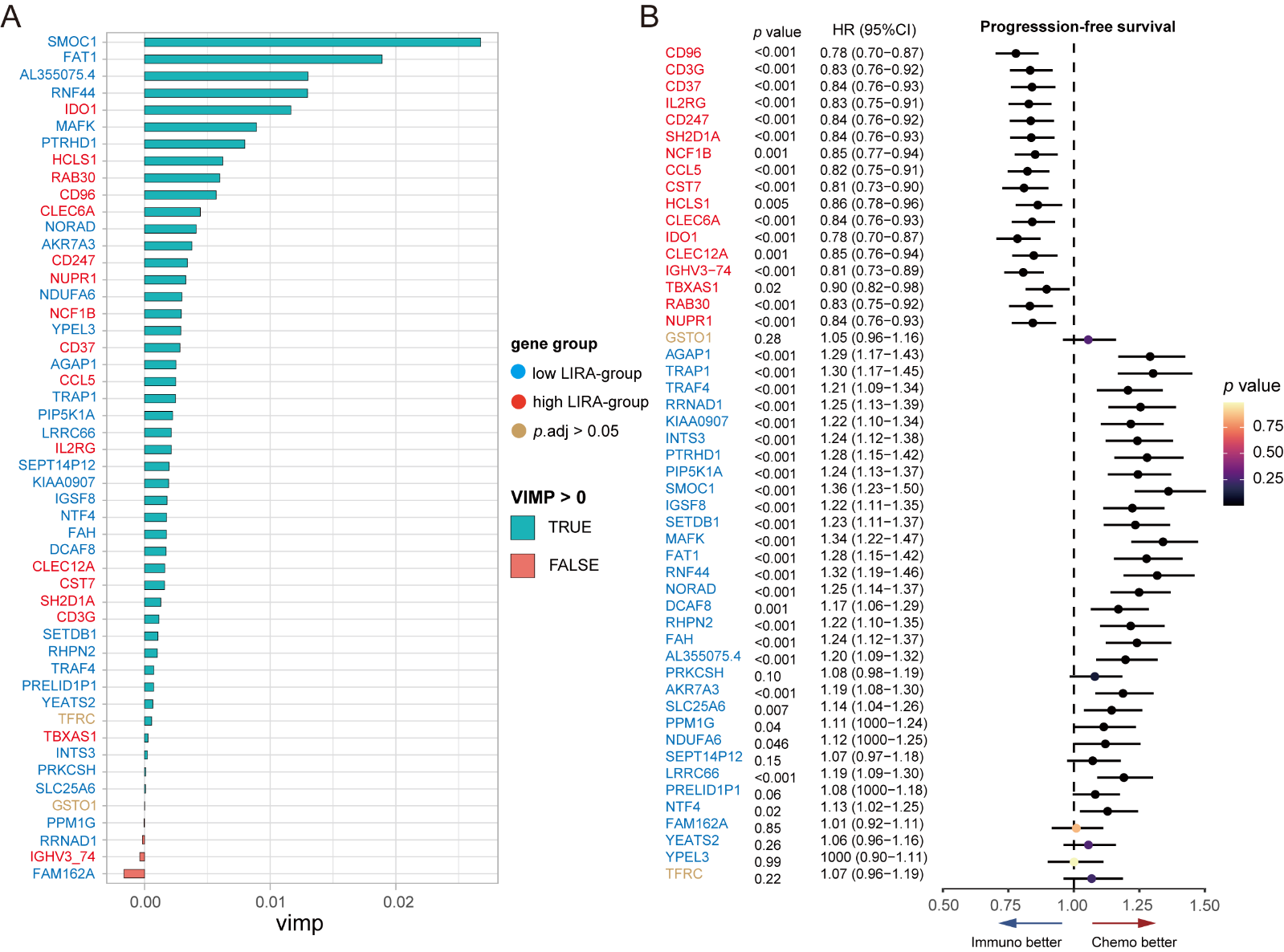
**Supplementary Figure 1.** Feature and survival analysis of model genes. A) The bar plot displays the relative importance of 50 model genes in LIRA's ability to make accurate predictions. B) A forest plot of 50 model genes correlated with PFS in the atezolizumab treatment is revealed. Red-colored genes are overexpressed in the high LIRA-score group. Blue-colored genes are overexpressed in the low LIRA-score group. LIRA, Lung cancer Immunotherapy Response Assessment; HR, hazard ratio; CI, confidence interval; VIMP, variable Importance.


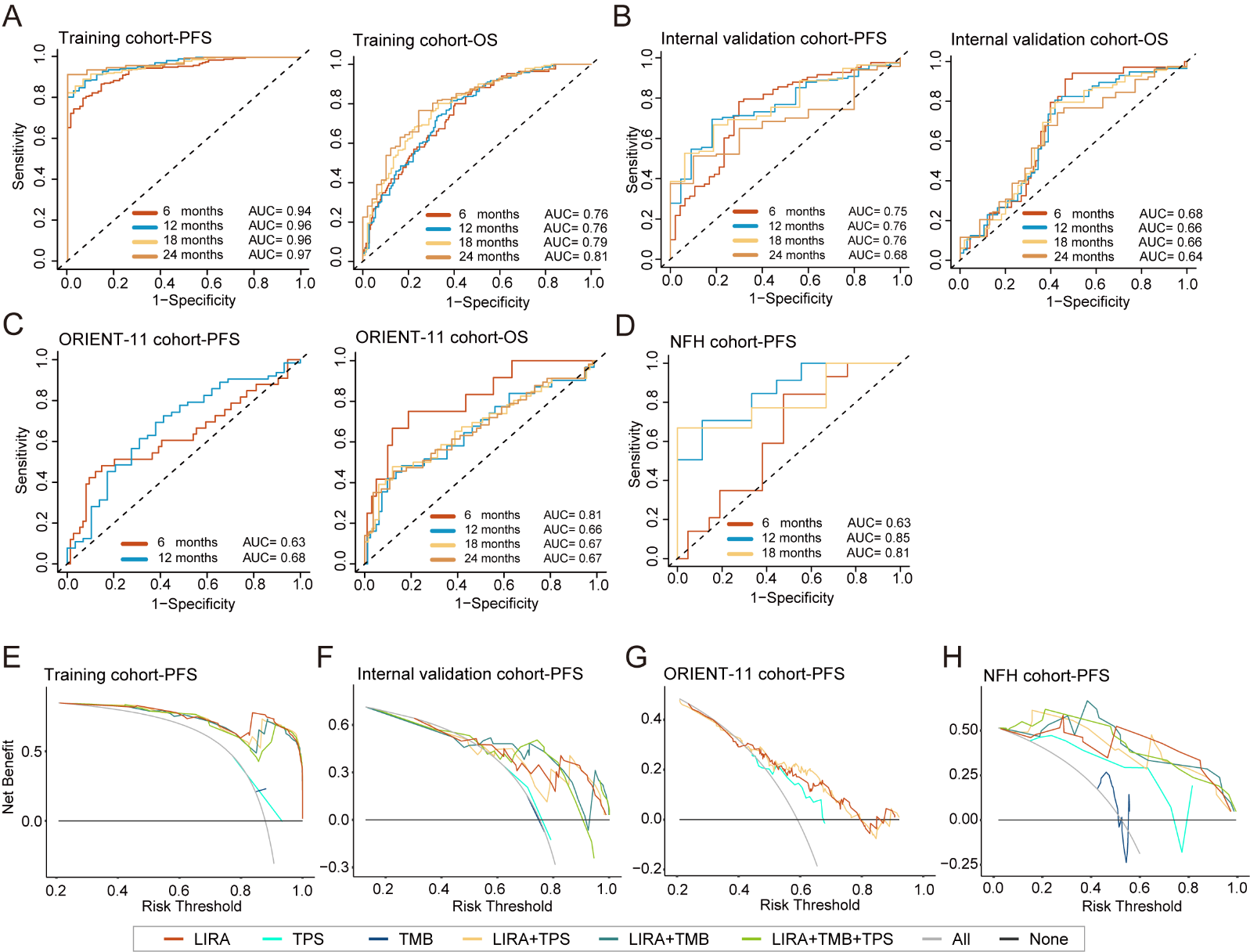
**Supplementary Figure 2.** ROC curves and survival analysis of LIRA in the training and validation cohorts. A–D) The time-dependent ROC curves and AUC for PFS and OS at four-time points (6, 12, 18, 24 months) in training (E), internal validation (F), ORIENT-11 (G) and in-house NFH (H) cohorts. E-H) Decision curve analysis for LIRA, tTMB/TMB, TPS, LIRA combining with TPS, LIRA combing with tTMB/TMB and LIRA combing with tTMB/TMB and TPS models in training (E), internal validation (F), ORIENT-11 (G) and in-house NFH (H) cohorts treated with ICIs treatment. ROC, receiver operator characteristic; AUC, area under curve; TPS, PD-L1 tumor proportion score; (t) TMB, (tissue) tumor mutational burden.


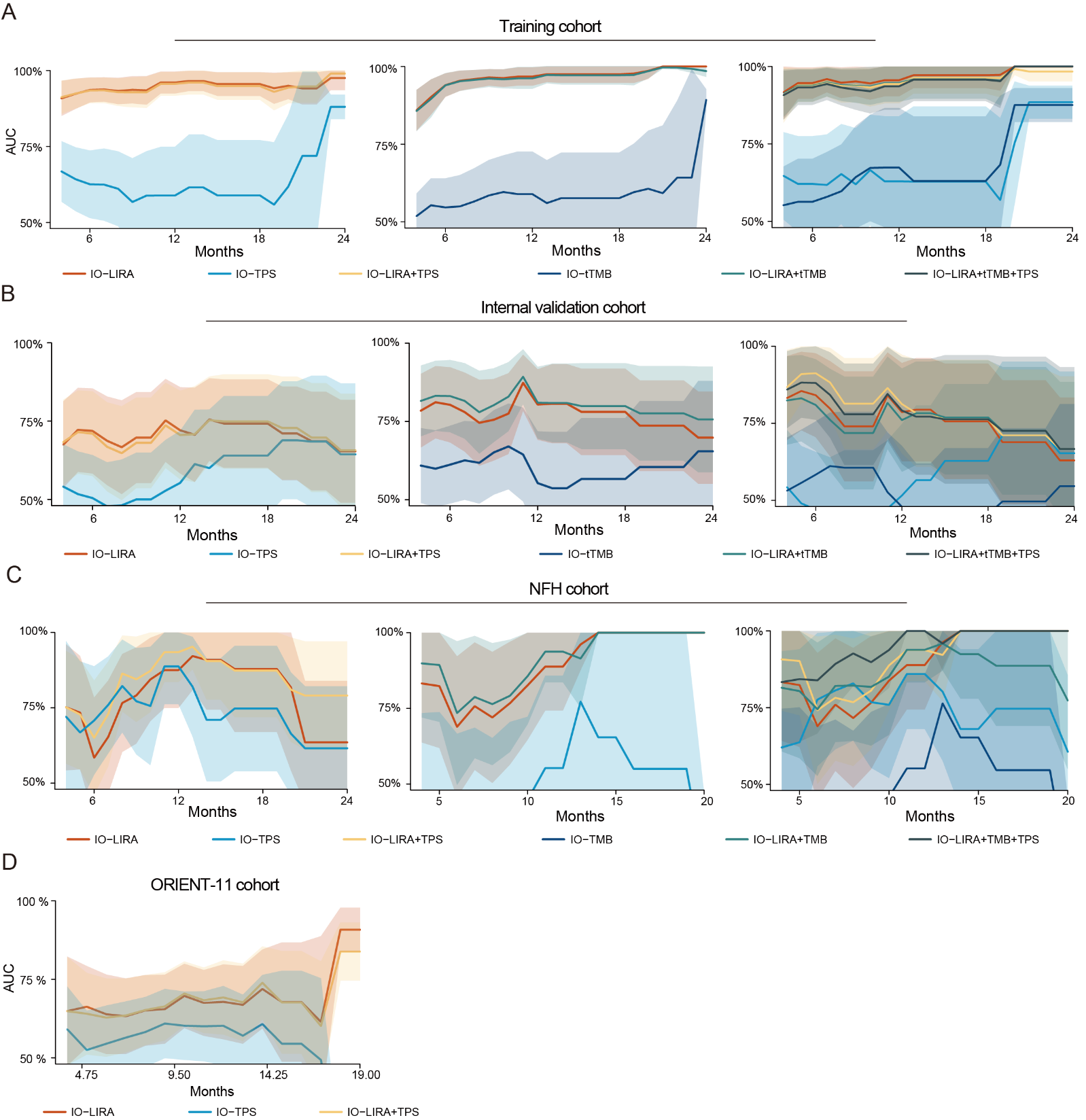
**Supplementary Figure 3.** Time-dependent AUC in the training and validation cohorts. A-D) Time-dependent AUC curves for LIRA (red), TPS (blue), tTMB/TMB (dark blue), LIRA combined with TPS (orange), LIRA combined with tTMB/TMB (green), and LIRA combined with TPS and tTMB/TMB (slate gray) in predicting the clinical benefit of patients treated with ICIs in the training (A), internal validation (B), in-house NFH (C), and ORIENT-11 (D) cohorts. IO, immunotherapy. TPS, PD-L1 tumor proportion score. (t)TMB, (tissue) tumor mutational burden.


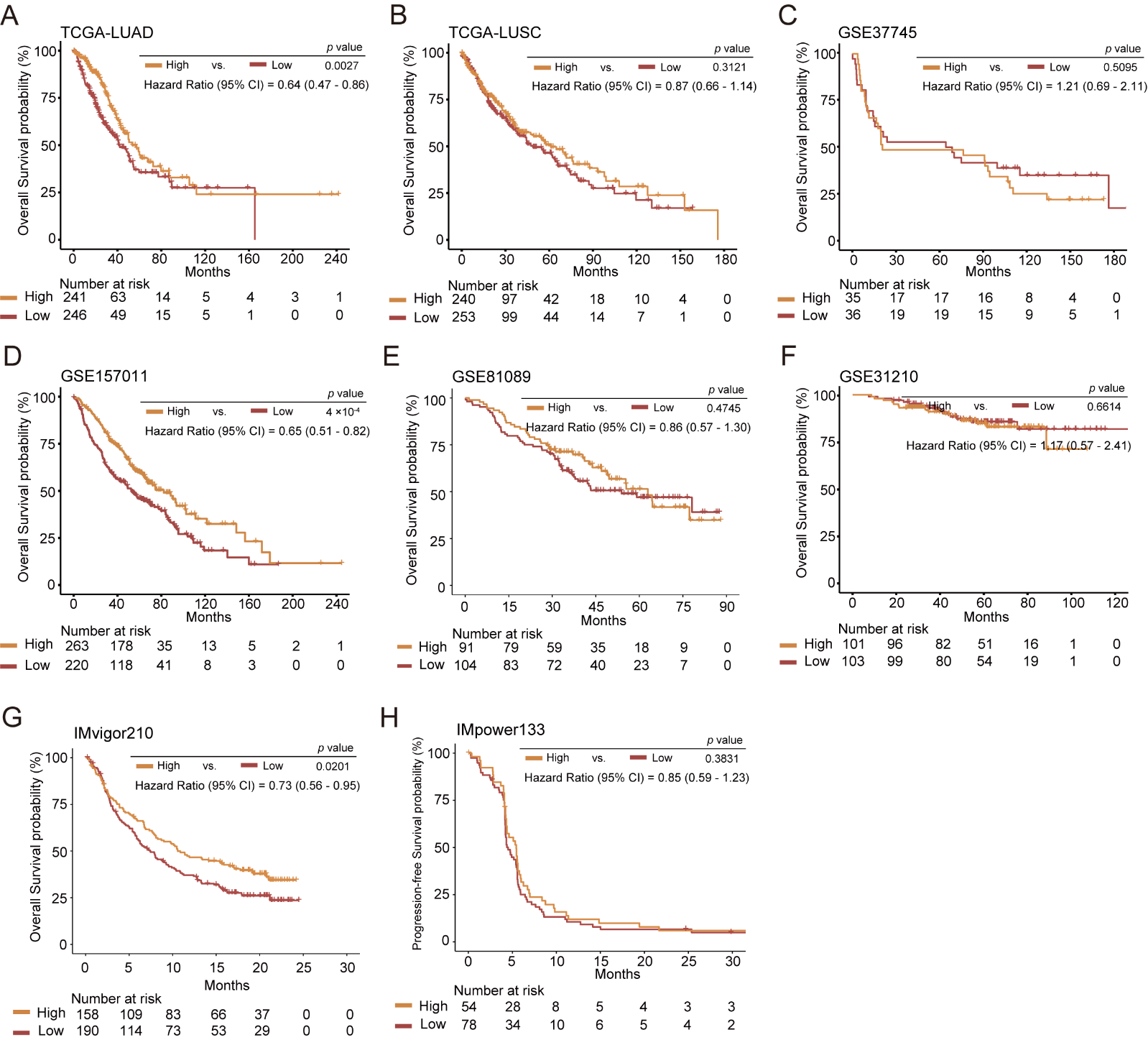
**Supplementary Figure 4.** Survival analysis in the NSCLC and non-NSCLC cohorts. A-H) Kaplan-Meier curves comparing survival outcomes (OS or PFS) between high and low LIRA-score groups in non-ICIs (A-F), IMvigor210 (G) and IMpower133 (H) cohorts.


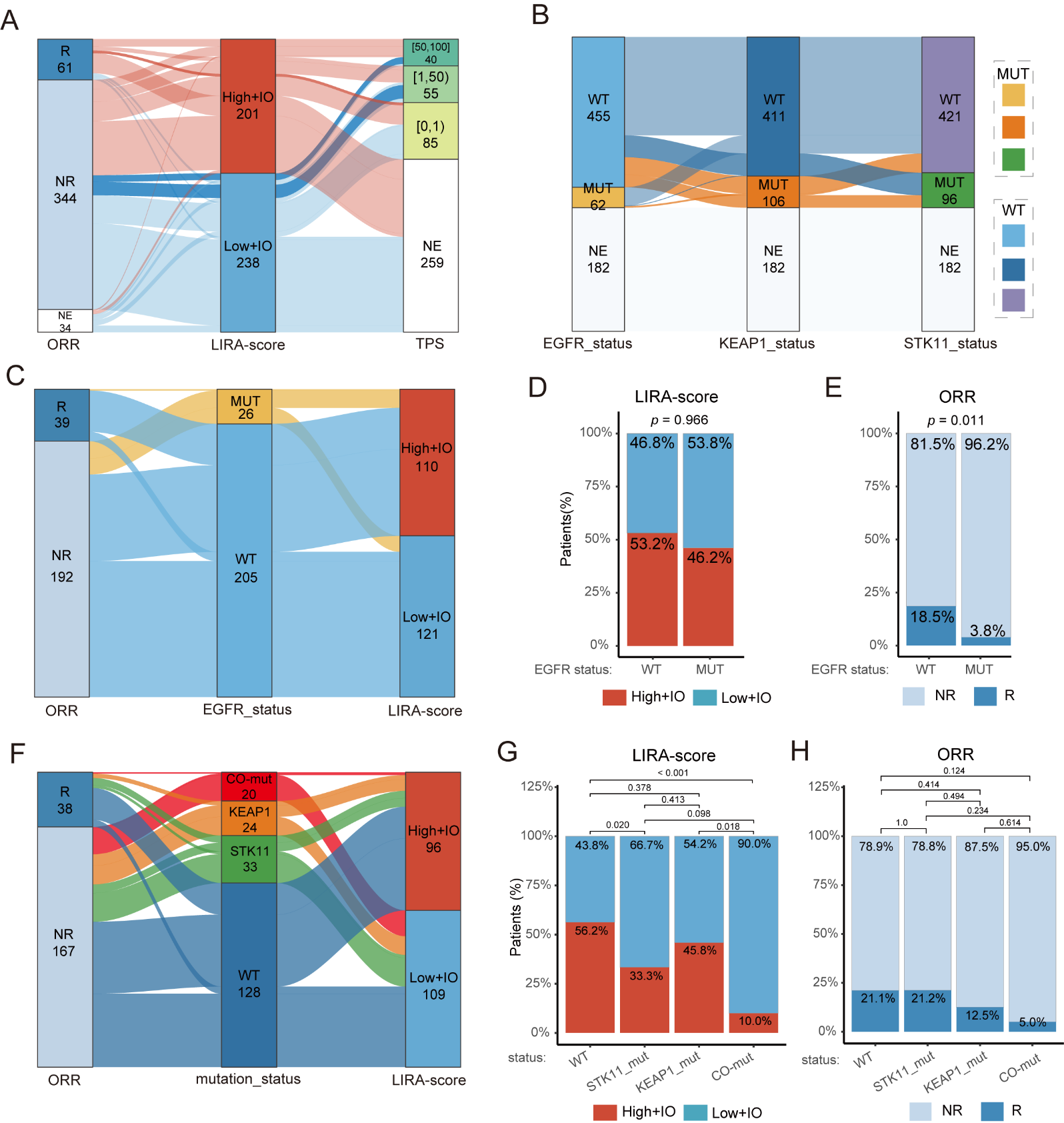
**Supplementary Figure 5.** Association of LIRA with clinical response and mutation status. A) Sankey plot showing the association among BOR, LIRA-score, and TPS. B) Sankey plot showing the association among *EGFR* status, *KEAP1* status, and *STK11* status. C) Sankey plot showing the association among BOR, EGFR status, and LIRA score. D-E) Percentage bar plot showing the relationship between *EGFR* status and LIRA (left) and BOR (right) respectively. F) Sankey plot showing the association among BOR, mutation status, and LIRA-score. G-H) Percentage bar plot showing the relationship between mutation status and LIRA (left) and BOR (right), respectively. BOR, best of response; ORR, objective response rate; CR, complete response; PR, Partial response; SD, Stable disease; PD, Progressive disease; R, response (CR, PR); NR, non-response (SD, PD); NE, not evaluate; Hist, histology; WT, wild type; MUT, mutation.


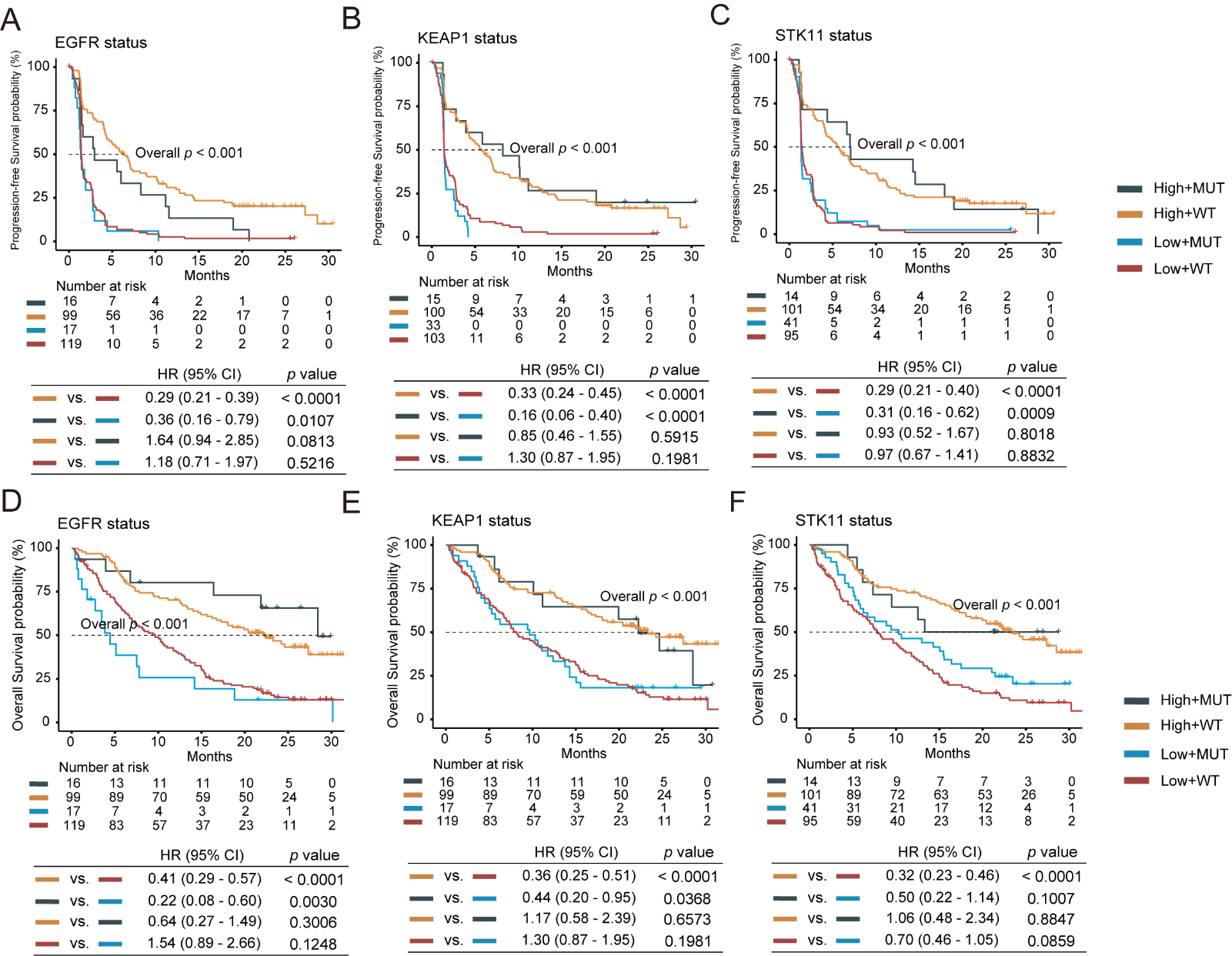
**Supplementary Figure 6.** LIRA predicts immunotherapy outcomes for patients with different mutation statuses. A-C) Kaplan-Meier curves comparing PFS between low and high-score groups based on mutation status in patients treated with ICIs. D-F) Kaplan-Meier curves comparing OS between low and high-score groups based on mutation status in patients treated with ICIs.


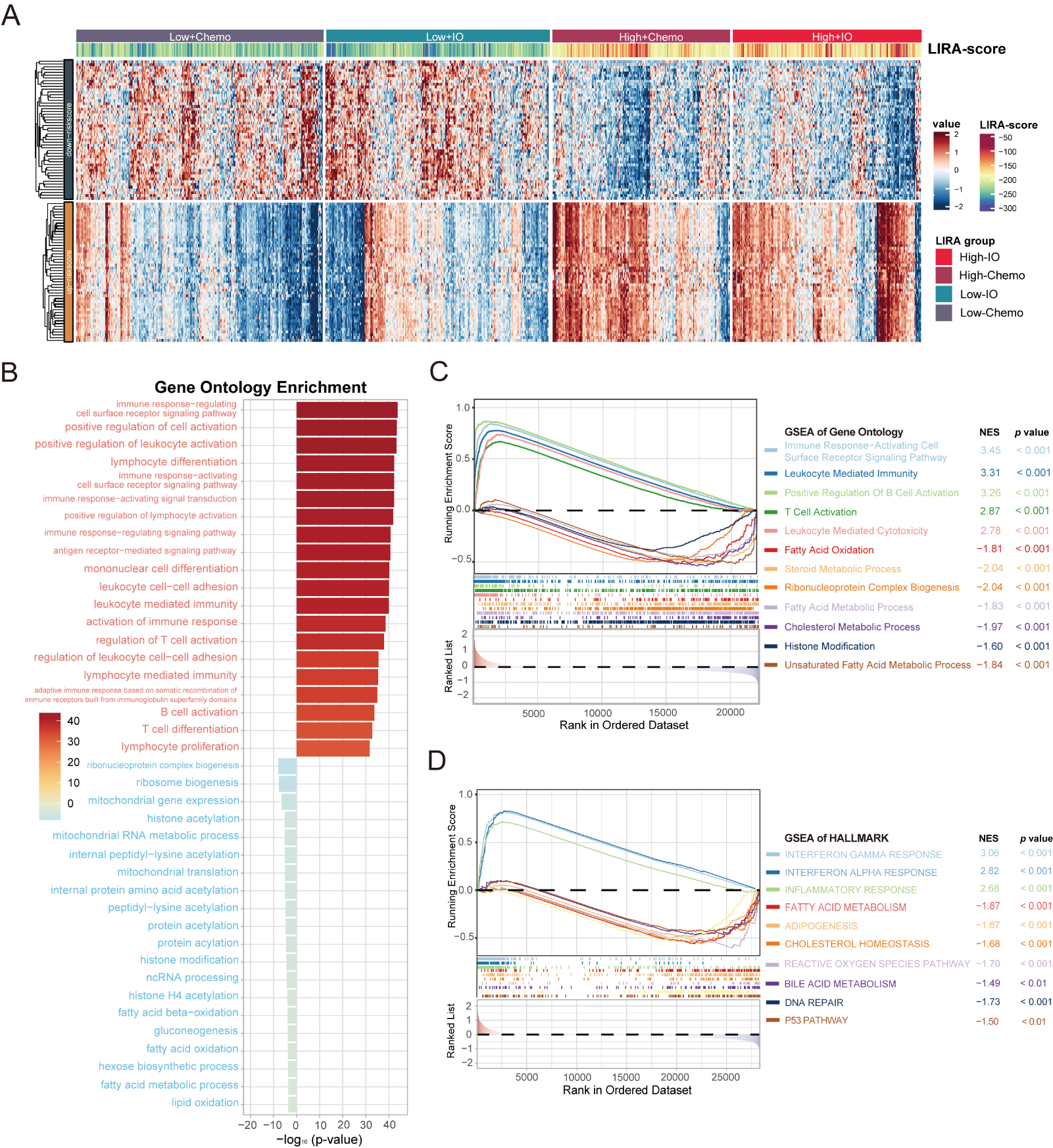
**Supplementary Figure 7.** Pathway enrichment analysis in two groups with atezolizumab treatment. A) Heatmap showing the genes overexpressed in low (n = 50 genes; statistic < 0, p.adj < 0.01) and high-score group (n = 50 genes; statistic > 0, p.adj < 0.01) in the four groups (n = 891 patients) based on LIRA-score and treatment (see Supplementary Table 7). Rows of the heatmap show the relative expression of genes. B) GO enrichment analysis of gene expression in high LIRA-score and low LIRA-score group. C-D) GSEA analysis displayed key pathways of GO and HALLMARK term enriched in high (up) and low (down) LIRA-score groups. GSEA, gene set enrichment analysis; GO, Gene Ontology; NSE, normalized enrichment score.


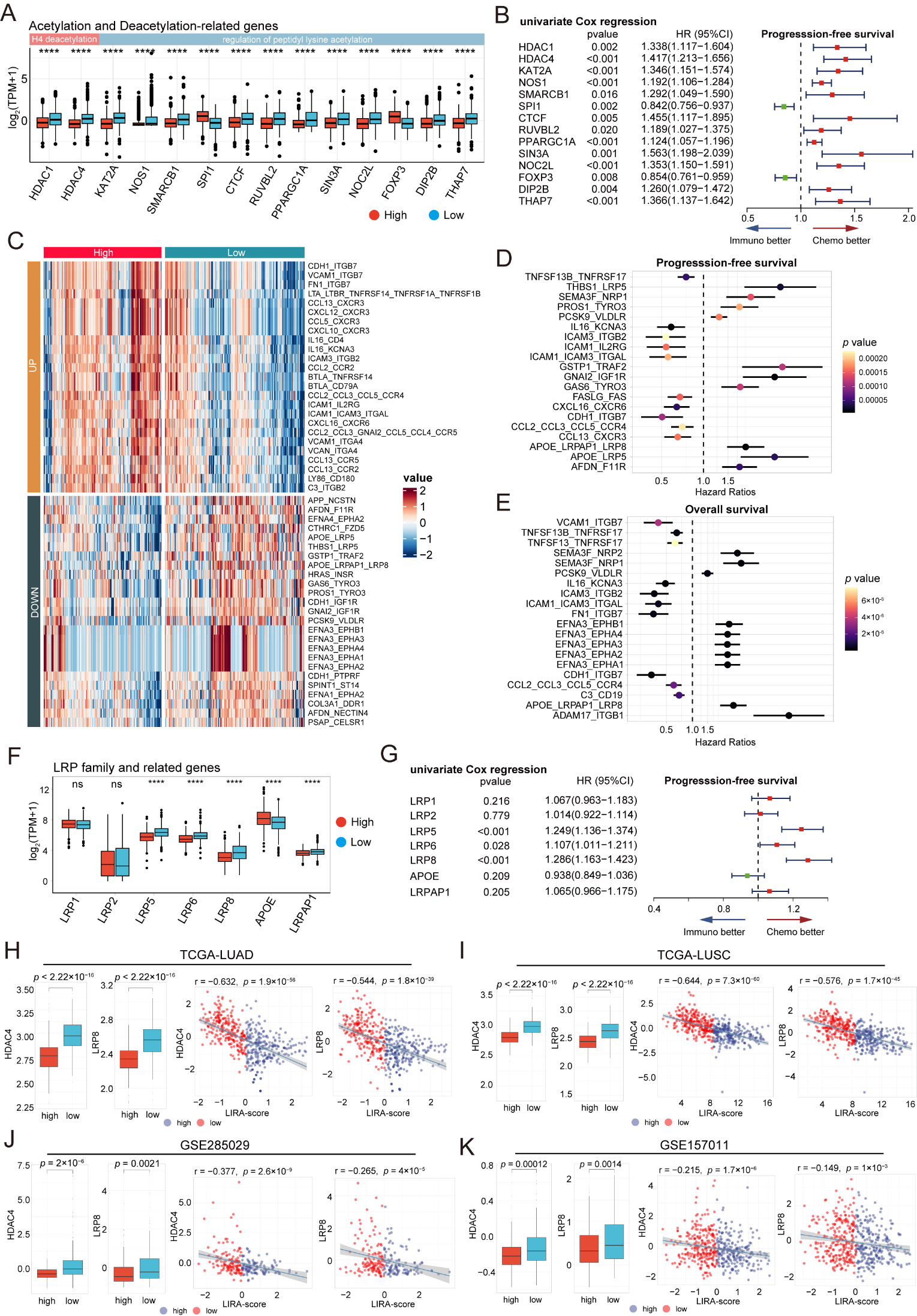


**Supplementary Figure 8.** Analysis of ligand-receptor pairs and key molecules. A) Boxplots showing log2(TPM+1) of acetylation and deacetylation-related genes in high and low score group with atezolizumab treatment. B) Forest plot showing the HRs, 95% CIs, and p values of acetylation and deacetylation related genes form based on univariate Cox analysis for PFS in patients receiving atezolizumab. C) Heatmap showing the interaction weight of LR in low and high LIRA-score groups with ICIs treatment. D-E) Forest plot showing the HRs, 95% CIs, and p values of ligand-receptor interaction score based on univariate Cox analysis for PFS (top) and OS (bottom) in patients receiving atezolizumab. F) Boxplots showing log2(TPM+1) of LRP family and related genes in high and low LIRA-score groups with atezolizumab treatment. G) Forest plot showing the HRs, 95% CIs, and p values of LRP family and related genes based on univariate Cox analysis for PFS in patients receiving atezolizumab. H-K) Boxplots and correlation plots showing the relationship between LRP8 and HDAC4 expression and the LIRA-score. P value in (A) (F) (H-K) was calculated using two-sided Mann Whitney U test. *p < 0.05, **p < 0.01, ***p < 0.001, ****p < 0.0001. ns, not significant compared to isotype group. Center line, box limits and whiskers represent the median, interquartile range and 1.5× interquartile range, respectively.


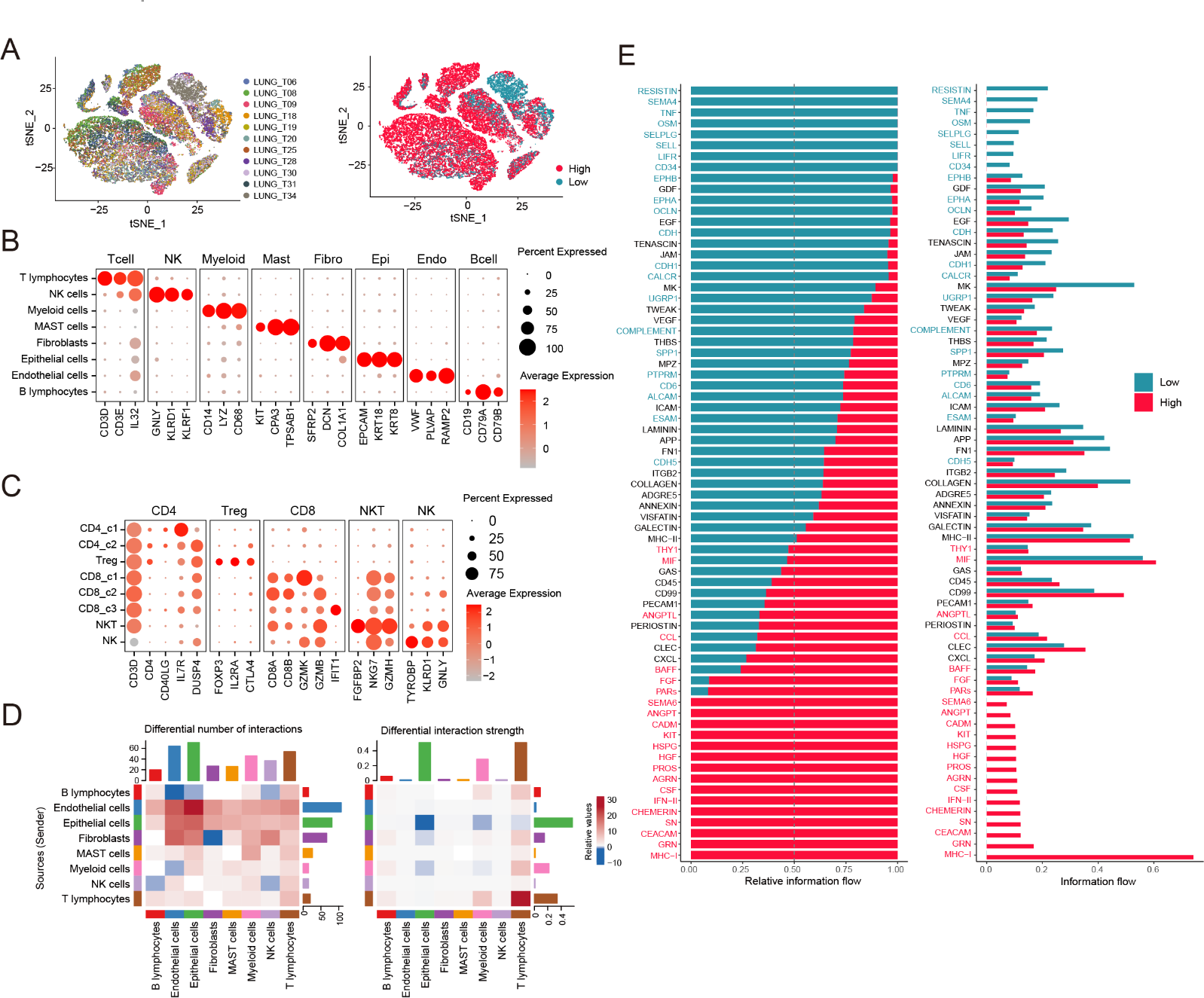
**Supplementary Figure 9.** Characteristics of cell-to-cell communication between high and low LIRA-score group. A) t-SNE plots show 41,779 single cells from 11 NSCLC patients. Points are color-coded by included patients (left) and LIRA-score (right). B-C) Marker genes used to annotate major cell types (B) and different T/NK subtypes (C). Size of dots indicates the proportion of cells expressing the selected genes, and intensity of color indicates the scaled average expression level. D) Heatmap showing the differential number of interactions and interaction strength between high and low LIRA-score samples. E) The comparative analysis of signaling pathway activities between high and low LIRA-score groups. The left panel shows the relative information flow of signaling pathways ranked by activity difference between high (red) and low (blue) LIRA-score groups. The right panel displays the absolute information flow of each pathway in the two groups.


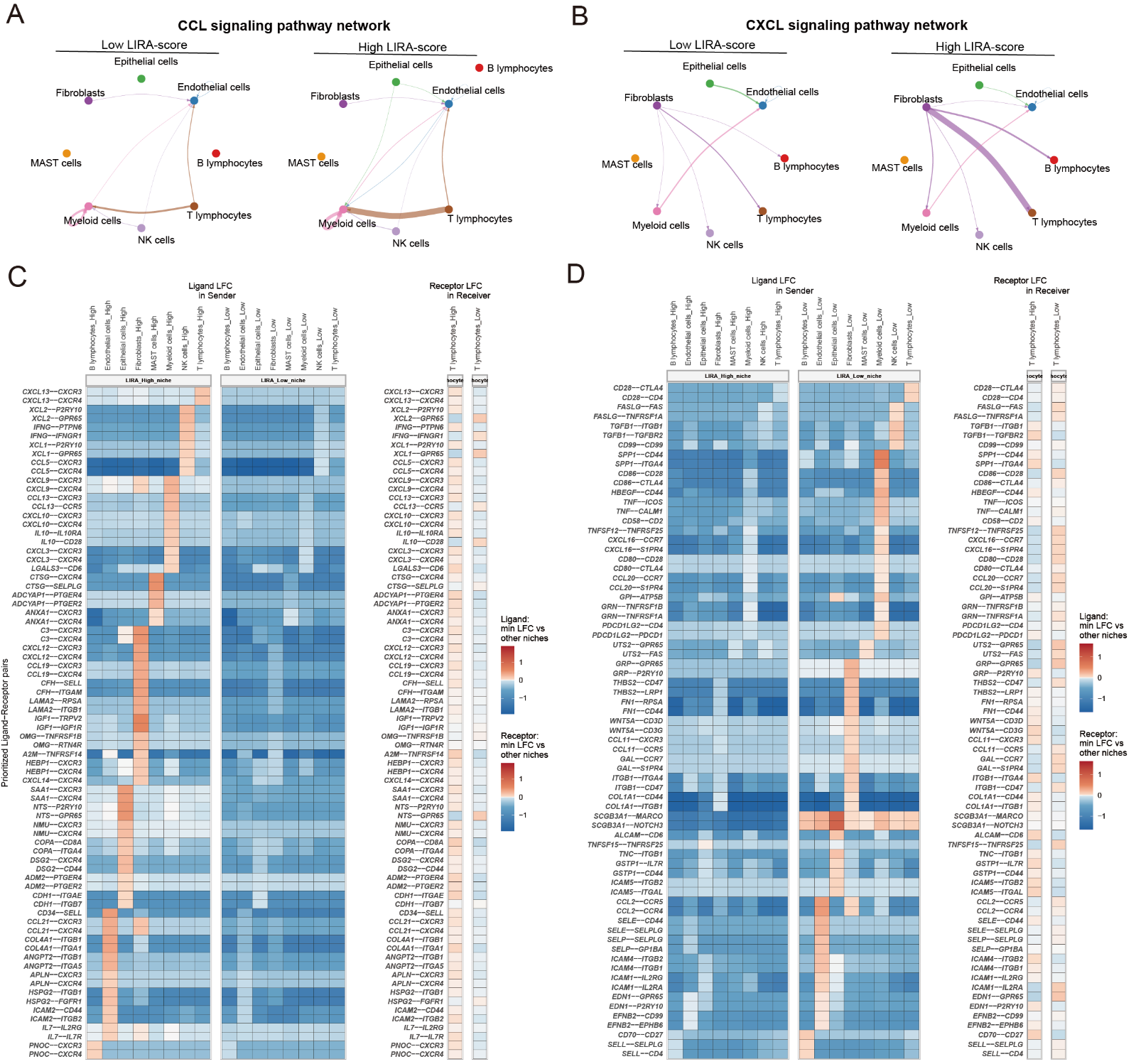
**Supplementary Figure 10.** Single-cell analysis of intercellular communication networks. A-B) Cell communication networks showing CCL (A) and CXCL (B) ligand-receptor interactions between different major cell types in low and high LIRA-score groups. C) Heatmap showing enriched ligand-receptor interactions between sender cells (left) and receiver T-lymphocytes (right) in the high LIRA-score group. D) Heatmap showing ligand-receptor interactions between sender cells (left) and receiver T-lymphocytes (right) in the low LIRA-score group. LFC, log fold change.
